# Transcriptomic profiling of the ovarian immune landscape reveals distinct macrophage subsets and activation of the NLRP3 inflammasome likely contributing to accelerated follicular atresia in classic galactosemia

**DOI:** 10.1186/s12964-026-02950-9

**Published:** 2026-05-30

**Authors:** Raghuveer Kavarthapu, Son Vo, Natalie Hanby, Christophe Vanpouille, Margaret Brunette, Mary E Soliman, Thang Pham, Hong Lou, Maria DeLa Luz Sierra, Jacqueline C Yano Maher, Veronica Gomez-Lobo

**Affiliations:** 1 Division of Pediatric and Adolescent Gynecology; Eunice Kennedy Shriver National Institute of Child Health and Human Development, National Institutes of Health, 10 Center Dr, Bldg10, Bethesda, MD 20892 USA; 2 BioTuring Inc, San Diego, California USA; 3 Division of Pediatric and Adolescent Gynecology, Children’s National Hospital, Washington, DC USA

## Abstract

**Background:**

Premature ovarian insufficiency (POI) is a complication that affects 80% of females with classic galactosemia (CG), an autosomal recessive metabolic disorder caused by mutations in the *GALT* gene. The immune system plays a critical role in normal ovarian physiology and in the development of pathological conditions, including POI. While previous animal studies have shown that accumulation of toxic galactose metabolites trigger cellular stress pathways, and may contribute to the disease’s complications, it remains unclear how these influence the ovarian immune landscape in CG patients.

**Methods:**

Here, we performed single-nucleus and spatial transcriptomic analyses on ovary biopsies from pre-pubertal girls with CG and compared with control ovary samples to explore the changes in ovarian immune milieu. We specifically investigated the gene expression profiles and altered signaling pathways in macrophages and endothelial cells. Mouse ovary cultures were treated with 50mM D-galactose for 48 h and immunohistochemical analysis was performed to evaluate the role of oxidative stress and activation of the NLRP3 inflammasome.

**Results:**

Our transcriptomic analysis reveals diverse subpopulations of macrophages inside the ovarian stroma. We noticed a marked increase in both monocyte-derived and tissue-resident macrophages, and expression of proinflammatory cytokine genes (*TNF*, *IL1B*, *CCL2*, *CXCL10*,* CXCL8*) in the CG ovary. Transcriptomic profiles of endothelial cells reveal upregulation of cell-adhesion molecules (*VCAM1*,* ICAM1*,* PECAM1*,* SELP*,* SELE)* and chemokines (*CCL21*,* CX3CL1*) which can modulate immune cell extravasation at the site of inflammation in the CG. IHC data revealed increased expression of NLRP3, CASP-1 and TNF-α expression in the ovaries of CG. Furthermore, D-galactose toxicity in mouse ovaries resulted in oxidative stress evident by increased expression of 8-OHdG and 4-HNE, and increased atresia of ovarian follicles indicated by cleaved-CASP3 expression. We found an increased expression of NLRP3, cleaved CASP1, activated GSDMD, cleaved IL-1β and TNF-α in the D-galactose-treated mouse ovaries, suggesting inflammasome-mediated pyroptotic cell-death and inflammation, which can eventually contribute to augmented follicular atresia.

**Conclusions:**

Our findings provide molecular insights into the proinflammatory immune response driven by oxidative stress-induced activation of the NLRP3 inflammasome pathway in the CG ovary. This study elucidates the plausible role of ovarian pyroptotic macrophages in the exacerbating the inflammatory microenvironment, which may explain the early onset of POI in CG patients.

**Supplementary Information:**

The online version contains supplementary material available at 10.1186/s12964-026-02950-9.

## Background

Galactosemia is a genetic disorder caused by a deficiency in enzymes essential for galactose metabolism via the Leloir pathway [1]. The most prevalent and severe type of galactosemia is classic galactosemia (CG), affecting approximately 1:50,000 infants in the USA [2]. Early symptoms in infants include poor growth, liver damage, kidney failure, jaundice, E. coli sepsis, and neonatal death [3–5]. This autosomal recessive disorder results from mutations in the GALT gene, which encodes the enzyme galactose-1-phosphate uridylyltransferase [3–5]. Due to lack of the GALT enzyme, CG patients accumulate high levels of metabolites such as galactose-1-phosphate and galactitol, contributing to the disease’s pathophysiology [5, 6].

Consequently, routinely screening in search of low GALT enzyme activity and elevated erythrocyte galactose-1-phosphate levels are critical for early diagnosis of CG in newborns [2]. While restricting dietary galactose intake can effectively relieve initial neonatal symptoms, long-term chronic issues often persist with varying severity. These issues include developmental delays, motor function abnormalities, speech and language acquisition delays, and early-onset premature ovarian insufficiency (POI) [3, 4].

POI is a condition in which normal ovarian function ceases due to lack of folliculogenesis or premature follicular loss, leading to hypergonadotropic hypogonadism in individuals under the age of 40 [7]. POI affects 80–90% of female CG patients regardless of treatment with a galactose-restricted diet [8]. Classic galactosemia patients with POI present with delayed or absent puberty, irregular menstruation, amenorrhea, and/or infertility [8] with elevated follicle-stimulating hormone levels, low anti-Mullerian hormone (AMH) levels, hypoestrogenism, and normal or increased luteinizing hormone levels [8]. While the onset of POI in CG is not well understood, prior research has shown normal follicle counts among CG patients at birth yet a sharp decline by early puberty [8–10]. We recently reported a significant reduction in mean follicle density and primordial follicle count in prepubertal girls with CG compared to controls [11]. These girls have predominantly primordial follicles and fewer transitional primordial follicles, with almost no primary follicles, suggesting impaired follicle activation and growth in addition to follicle depletion. Further, the AMH serum levels were found to be very low to undetectable levels in CG patients [11].

The GALT-KO mouse model demonstrated limitations in growth, diminished motor abilities, and subfertility in adult females. However, there was no notable decrease in primordial follicles in younger mice at postnatal days 14 and 30 [12]. Consequently, the animal models of CG may not effectively mimic POI as commonly seen in human CG cases. D-galactose toxicity and its metabolites were shown in mice to induce ovarian follicle atresia and reduce oocyte quality through significant enhancement in the production of ROS (oxidative stress) leading to POI [13–15]. Our recent findings from single-nucleus RNA sequencing (snRNA-seq) analysis of the human ovary from prepubertal girls with CG revealed activation of oxidative stress and ER-stress pathways genes resulting in DNA damage, autophagy and apoptosis of ovarian follicles [16]. In conjunction with the gonadotoxic effects of elevated galactose levels and its metabolites, we hypothesize that changes in the immune system response can also contribute to ovarian dysfunction and depletion of follicles observed in affected individuals with CG. It has been well-established that the immune system plays a crucial role in regulating normal ovarian function by influencing various stages of follicular development, ovulation, and corpus luteum formation through the release of cytokines and chemokines [17–19]. In human ovaries, CD68+ macrophages are the most abundant immune cell population, and are predominantly localized in corpora lutea, stromal tissue and near vascular cells [20, 21]. Macrophages promote ovarian vascularization, primordial follicle activation, follicle development, progesterone production, luteinization and luteolysis [20, 21]. Ovarian macrophages secrete an array of cytokines (TNF-α, IFN-γ, G-CSF, IL-1, IL-6, IL-8, MCP-1, and GM-CSF) which have been implicated in the regulation of various aspects of ovarian function [20, 21]. With reproductive aging, it has been shown that the M1 and M2 macrophage subpopulations become increasingly prevalent [22, 23]. The M1 macrophages play a pro-inflammatory role by secreting high levels of cytokines including IL-1, IL-6 and TNF-α, exacerbating granulosa cell apoptosis and follicular depletion [22–24]. The M2 subset is involved in tissue repair and resolution of inflammation by facilitating the deposition of ovarian extracellular matrix (ECM) and the development of fibrosis by releasing elevated amounts of TGF-β, FGF, and PDGF [22–24]. Recent studies have shown that inflammatory aging may play a significant role in the pathogenesis of POI in women caused by exogenous and endogenous factors [25]. Therefore, the focus of our study aimed at understanding the role of the immune system, specifically macrophage regulated immune response in the POI condition observed in CG patients.

Here, we investigated the molecular and cellular changes in the ovarian immune environment that may impact the ovarian function contributing to early onset of POI in prepubertal girls with CG. Using the 10x Genomics platform, we created a comprehensive single-nucleus transcriptomic and spatial landscape of human ovaries from prepubertal girls diagnosed with CG and compared it with that of a control group. We first identified and characterized the immune cell population in the ovarian stroma. Second, we evaluated transcriptomic changes in macrophages and possible immune-related signaling pathways prompting the inflammatory response. Third, we identified four different distinct subpopulations of ovarian macrophages and focused on two subtypes that corresponded to tissue resident macrophages and monocyte-derived macrophages to define their transcriptional profiles. Using mouse whole ovary culture model, we demonstrated that D-galactose toxicity induces oxidative stress, which in turn activates the inflammasome pathway, triggering inflammation and pyroptotic cell-death of ovarian follicle. Finally, we also explored the role of endothelial cells in modulating the immune response through the recruitment of immune cells to the site of inflammation. Our findings elucidated the mechanisms underlying follicle depletion and ovarian dysfunction in GC, and are crucial for the development of effective therapeutic strategies to preserve ovarian function and prolong female reproductive health in CG patients.

## Methods

### Ethics and human sample collection

All the studies involving human subjects were approved and conducted in accordance with the Institutional Review Board (IRB) of Eunice Kennedy Shriver National Institute of Child Health and Human Development (NICHD; protocol numbers IRB000106, IRB00715) at the National Institutes of Health and at Children’s National Hospital in Washington, DC (IRB protocol numbers Pro00010699, Pro00016433). All patients provided signed informed consent to donate the ovary tissue for research purposes. Ovarian tissue biopsies were collected from three prepubertal girls (age group five to ten years) diagnosed with CG disorder who have diminished number of ovarian follicles (Suppl Table S1) and underwent laparoscopic unilateral oophorectomy for fertility preservation at NICHD, National Institutes of Health, Bethesda, USA. Due to the invasive nature of collecting ovarian tissue, the ability to obtain tissue biopsies from healthy prepubertal girls precludes healthy controls due to IRB ethical considerations. Hence, children with solid tumors (non-systemic cancer) away from reproductive system which require gonadotoxic therapy were deemed the best controls. For this, we utilized ovaries collected from three prepubertal female children (Supplementary data Table S1) who also underwent unilateral oophorectomy prior to gonadotoxic therapy at Children’s National Hospital in Washington, DC, conducted under a similar protocol. All control ovary samples were confirmed negative for histopathological abnormality by a pathologist, and these patients have normal mean follicle density (with large primordial follicle pool, primary and growing follicles) and normal levels of serum AMH. While CG patients have significantly fewer primordial follicles, no pre-antral follicles, and undetectable levels of serum AMH compared to controls [11]. Collected ovarian tissue biopsies were flash frozen for snRNA sequencing and fixed in 4% paraformaldehyde or modified Davidson’s fixative for histological evaluation, ST mapping, and immunohistochemistry. Patient demographics showing age, diagnosis, primordial follicle count, and mean follicle density are described in Table S1.

### Single-nucleus RNA sequencing and data analysis

#### Preparation of single nucleus suspensions from ovary tissue

For frozen ovarian tissue samples, snRNA-seq approach is a promising method to explore the cellular diversity and molecular complexity of ovarian cells at single cell level. Approximately, 30-40 mg of flash-frozen ovarian biopsy was used to isolate nuclei according to the modified method from the 10X Genomics Chromium Nuclei Isolation kit (dx.doi.org/10.17504/protocols.io.6qpvr4jb2gmk/v1). Ovarian tissue samples were lysed with detergent lysate buffer by mechanically grinding with a pestle and incubated on ice for 10 min. The lysate was first filtered through 70 μm cell strainer, then again with 40 μm cell strainer, and the homogenate was collected. The nuclei were purified by washing them in a nuclei suspension buffer followed by sucrose density gradient centrifugation. The nuclei concentrations and quality were evaluated using a CellDrop cell counter as per the manufacturer's instructions (DeNovix). 

#### SnRNA-seq library preparation and sequencing

Following the manufacturer’s instructions, snRNA-seq libraries were built from nuclei extracted from ovarian tissue using the 10x Genomics Chromium Next GEM Single Cell 3ʹ Kit v3.1 (10X Genomics, PN-1000268). In summary, a total of 8,000 freshly prepared nuclei were loaded onto a Chromium GEM-X chip, designed for creating single-cell gel bead emulsions with the 10x Genomics Chromium controller. Subsequently, cDNA amplification was carried out, and libraries were constructed following the manufacturer's guidelines. The libraries underwent quantification and quality evaluation using a Bioanalyzer (Agilent). Finally, the libraries were combined and sequenced on an SP flow cell (paired-end 100 bp) utilizing the Illumina NovaSeq 6000 platform. 

#### Raw data processing and quality control

The raw sequencing reads (FASTQ format) of each sample were processed and aligned to the GRCh38 human reference genome using the Cell Ranger V4.1 pipeline with default parameters. Alignment of sequencing reads, barcodes and UMI counting were performed to obtain a gene barcode matrix file (gene counts per nucleus) using the Cell Ranger count. For downstream bioinformatic analysis, we used the BioTuring platform (BioTuring Inc.) where filtering of nuclei, clustering and cell type annotation were performed. For downstream bioinformatic analysis, we used BioTuring platforms (BioTuring Inc.) including BBrowserX, Lens, and BioStudio for filtering low quality nuclei, clustering, annotating cell types, and other customized analyses. Low quality nuclei with fewer than 200 detected genes and fewer than 500 transcriptomic counts were excluded. 

#### Clustering, identification and annotation of cell types

After quality control, normalization was performed across all cells with a library size of 10000, followed by log1p transformation. The batch effect between six samples was removed using the harmony package. Subsequently, we performed UMAP with n_neighbors=30 and Louvain clustering with resolution = 1.0 on the harmony latent space. To detect the differentially expressed genes for each cell cluster, “FindAllMarkers” Seurat function was used based on Wilcoxon rank sum test with default parameters. We then classified/annotated different ovarian cell types (granulosa cells, oocytes, endothelial cells, muscle cells, epithelial cells, immune cells, and stromal cells) based on canonical marker genes identified for each cluster that were previously reported in single-cell human ovary atlas [26–28]. To annotate immune and macrophage cell subtypes, we first isolated the target clusters, then re-performed the same procedure of batch correction, dimensionality reduction (n_neighbors=10), and clustering annotation. 

#### Differential gene expression analysis

To identify differentially expressed genes (DEGs) between CG and control groups, we used Seurat FindMarkers function with the Wilcoxon rank-sum test. DEGs with log2FC ≤ –0.5 or ≥ 0.5 and FDR (Padj value) < 0.05 were selected for downstream analysis such as gene ontology (GO) and IPA enrichment analysis.

### Spatial transcriptomics and data analysis

#### Spatial transcriptomic experiment

Visium ST experiment was performed on the same CG and control patient ovarian tissue samples used for snRNA-seq as per the manufacturer’s protocol (10x Genomics). Briefly, 5 μm thick modified davidson’s-fixed paraffin-embedded ovary sections were mounted onto a positively charged slide followed by staining with hematoxylin and eosin. Slides were mounted in 75% glycerol and brightfield images were taken using a Keyence microscope. The tissue sections were then transferred immediately onto 6.5 × 6.5mm capture areas on a VisiumHD gene expression slide using Visium CysAssist (10x Genomics, PN-1000187). The tissue sections were then subjected to permeabilization followed by reverse transcription for cDNA synthesis. Subsequently, the cDNA amplification was performed, and spatial libraries were constructed as per manufacturer’s instructions (CG000239 Rev H) using Visium spatial library construction kit (10x Genomics, PN-1000187). The libraries were pooled and paired-end sequencing was performed on SP flow cell using an Illumina NovaSeq 6000 platform. 

#### ST data processing

Spatial barcodes were used to associate the reads back to the tissue section images for Visium gene expression mapping. Space Ranger v3.0 from 10x Genomics was used to process raw sequencing output files and histology images of ovary sections to generate feature-barcode matrices for each sample. In-tissue ST spots were identified and retained based on the histological images. We processed feature-barcode files using the Seurat package on BioTuring Lens platform for downstream ST analysis to obtain information about the number of spatial spots, median genes and reads per spot. 

#### Clustering and cell type annotation of ST data

For ST cell type annotation, we employed SCTransform from Seurat with "glmGamPoi" method on the raw expression data and then applied Harmony package to remove the batch effect between six spatial slides. Dimensional reduction and clustering annotation were performed with UMAP (n_neighbors=30) and Louvain (resolution=1.0) on the Harmony latent space, respectively. The clusters were annotated based on cluster‐specific marker genes identified using the ‘FindMarkers’ function with default parameters, including the Wilcoxon rank sum test and canonical marker gene expression reported in the single-cell human ovary atlas. 

#### Integration of scRNA‐seq and ST data

We used a deconvolution approach to spatially map different cell types of human ovary, where marker genes from our snRNA-seq dataset served as a reference. To gain better resolution on the ovary ST data, we employed Cell2location (a Bayesian model for spatial mapping of cell types which estimates cell abundance by decomposing spatial data into reference expression signatures of cell types) with our snRNA-seq dataset from the same patient cohort as the reference to perform cell-type deconvolution [29].

The model was trained on a GPU with parameters: max_epochs=30000, batch_size=None, train_size=1. The spatial distribution of a specific cell type was visualized using the Seurat function SpatialFeaturePlot. We then performed differential expression analysis on the macrophage ST spots between CG and control groups using FindMarkers function with the Wilcoxon rank-sum test to obtain DEGs.

### Functional enrichment and causal network analysis

GO enrichment analysis was performed using web-based Enrichr tool or cytoscape software to obtain significantly enriched biological processes. Pathway enrichment and Upstream regulator analyses were performed on the DEGs using Qiagen Ingenuity Pathway Analysis (IPA) platform to identify significantly enriched canonical signaling pathways, biological functions, and upstream transcriptional regulators. Each gene identifier was mapped to its corresponding gene object in the IPA knowledge base. We applied default settings using Fisher’s exact test to determine the significantly enriched canonical pathways or biological function with *P* < 0.05. Upstream regulator and causal network analyses tool by IPA was used to identify potential upstream regulators (transcription factors, kinases, growth factors/receptors) and their corresponding downstream target genes from the DEGs list. Causal network analysis provides mechanistic gene regulatory networks that could explain the observed dynamic changes in gene expression in a canonical signaling pathway(s) or biological function. AUCell scores (gene set activity scores) for the enriched biological processes/pathways were obtained at the single-cell level based on the expression of gene sets from the human MSigDB database (https://www.gsea-msigdb.org/gsea/msigdb/human/genesets.jsp?collection=GO:BP and Qiagen GeneGlobe canonical pathways database (https://geneglobe.qiagen.com/at/knowledge/pathways) using the AUCell package [30].

### Cell-cell communication analysis using CellChat

To study and visualize the cell-cell communications changes across cell types, the R package “CellChat” was used with standard workflow. The human ligand-receptor interaction database CellChatDB was used for the subsequent analysis, and the “computeCommunProb” function from CellChat was employed to calculate communication probability and predict major incoming and outgoing signals across the signaling pathways. We compared the total number of ligand-receptor interactions and interaction strengths between interacting cell groups and visualized them with circle plots and heatmaps. We used netAnalysis_signalingRole_heatmap function to visualize the signaling strength in each cell type.

### Immunohistochemistry (IHC)

Paraffin embedded ovarian sections of 5 μm thickness were deparaffinized and rehydrated in a graded series of ethyl alcohol and distilled water. Antigen retrieval was performed for 20 min with universal epitope recovery buffer (Electron microscopy sciences, PA, USA). IHC was carried out using ImmPRESS Excel Amplified HRP polymer/ ImmPRESS HRP Universal PLUS polymer staining kits (Vector Laboratories) as per the manufacturer’s protocol. Tissue sections were incubated in BLOXALL reagent for 10 min and washed with PBS for 5 min. Blocking was performed with 2.5% horse serum in PBS for 30 min. After blocking step sections were incubated overnight with 1:250 dilution of respective primary antibodies such as CD68 (cell signaling technology, cat# 97778), CD86 (cell signaling technology, cat# 91882), CD80 (cell signaling technology, cat# 54521), CD163 (cell signaling technology, cat# 93498), TNF-α (cell signaling technology, cat# 3707), and cleaved-CASP3 (cell signaling technology, cat# 9664), cleaved-CASP1 (cell signaling technology, cat# 89332), 4-HNE (Santa Cruz Biotechnology, cat# sc- sc-130083), 8-OHdG (Santa Cruz Biotechnology, cat# sc-393871), NLRP3 (Santa Cruz Biotechnology, cat# sc-518122), cleaved GSDMD (cell signaling technology, cat# 39754), and cleaved IL-1β (cell signaling technology, cat# 63124).

After incubation the sections were washed with PBS and incubated with the amplifier antibody for 15 min. Subsequently, sections were washed with PBS with 0.1% tween-20 and incubated with anti-goat HRP polymer reagent for 30 min. The sections were washed, diaminobenzidine (DAB) reaction was performed to develop the brown color for visualization. Sections were counterstained with hematoxylin, washed in 100% ethanol, and mounted using VectaMount medium (Vector Laboratories). The immuno-stained sections were imaged using an EVOS M5000 microscope (Thermo Scientific).

### Mouse ovary culture and D-galactose treatments

Ovaries were collected from 6 to 8 weeks C57BL/6 mice (*n* = 3) in growth medium containing αMEM (Invitrogen, CA) supplemented with 5% FBS, 1% Insulin-Transferrin-Selenium (Sigma-Millipore, MA), and penicillin-streptomycin. Each ovary was placed on the top of cell culture inserts (0.4 μm) floating on a growth medium for 1 day in a CO2 incubator at 37 °C. Treatments were carried out by adding 50mM concentration of D-galactose to the culture medium for 48 h. After treatment the ovaries were fixed in modified Davidson’s fixative and processed to obtain FFPE ovarian sections to perform immunostaining to evaluate the expression of cleaved-CASP3 for cell-death, GSR for oxidative stress, NLRP3 and GSDMD/GSDME for inflammasome pathway and TNF-α as an inflammation marker. Mouse experiments were performed in accordance with the guidelines established by the National Institute of Child Health and Human Development Animal Care and Use Committee. Adult female mice (6–8 weeks) were maintained at 22 °C in a pathogen-free, light-controlled environment with an alternating light–dark cycle and ad libitum access to water and food.

### Statistical analysis

All the data for continuous variables are expressed as the median and interquartile range using a violin plot. Statistical analysis was performed using GraphPad Prism version 9.2. Statistical differences between the two groups were performed using a two-tailed unpaired t-test, and *P* value < 0.05 was considered statistically significant.

## Results

### snRNA-seq profiling and spatial transcriptomics of human ovary from prepubertal girls

**Fig. 1:**
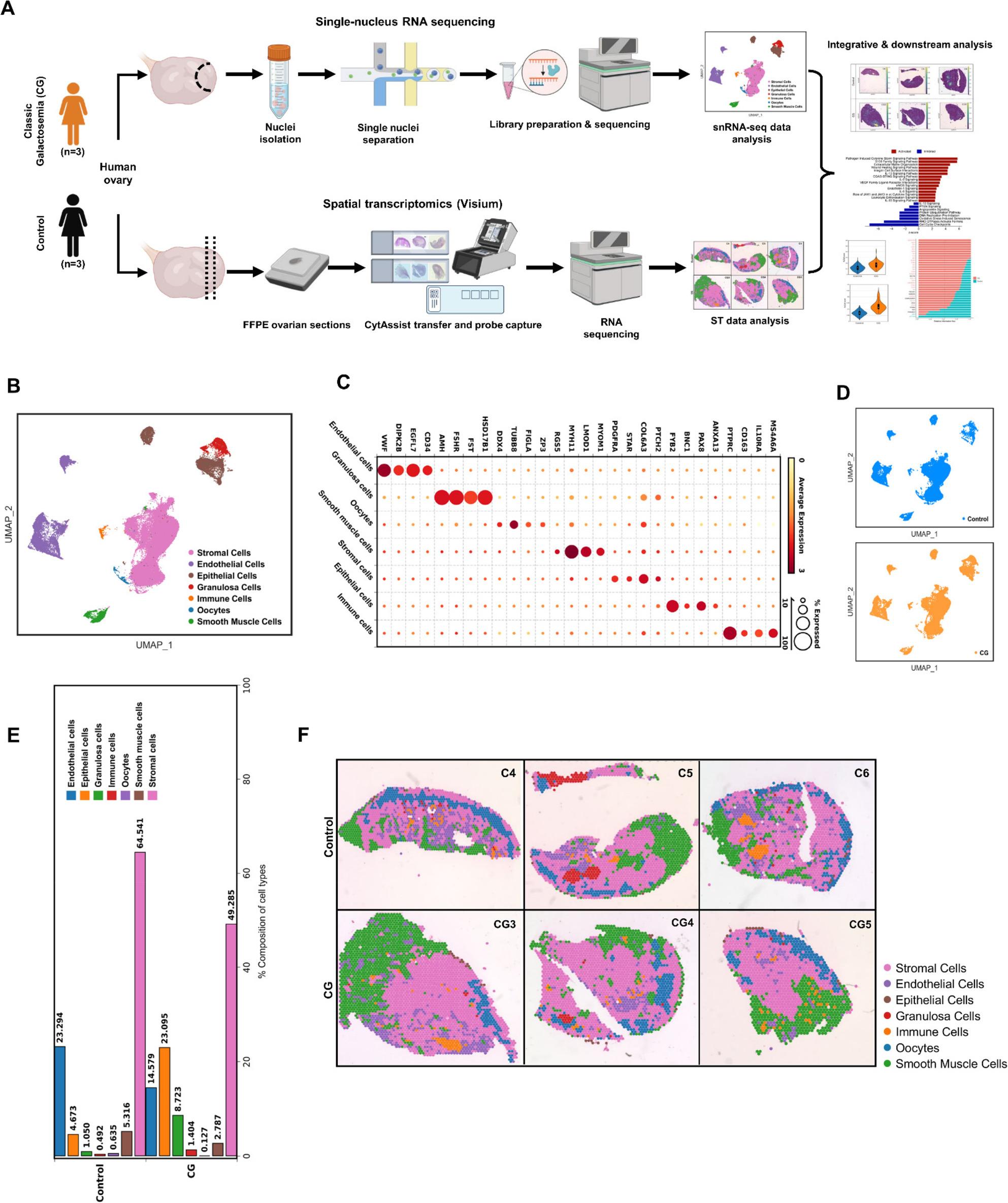
Single-nucleus and spatial transcriptomic landscape of human ovaries from prepubteral females. **A** Schematic diagram showing the experimental workflow for snRNA-seq and ST analysis of human ovaries from CG and control groups. **B** UMAP plot showing the distribution of the seven major distinct cell types of human ovary from all the samples based on snRNA-seq data. **C** Bubble plot showing the specific canonical marker genes unique for different cell types identified in the human ovary atlas. **D** UMAP plot showing distribution of nuclei in the CG and control groups. **E** Box plot represents the percentage composition of each cell type in CG and control groups . **F** Spatial plots portraying the seven major cell types identified by ST analysis

We utilized 10x Genomics Chromium GEM-X and Visium-ST platform to generate a comprehensive single-nucleus and spatial transcriptomic profiles of human ovary from prepubertal girls diagnosed with CG. The overview of the study design for the human ovarian transcriptomic atlas is shown in Fig. 1A. After quality control and normalization (Figure S1A and S1B), snRNA-seq data from all the samples resulted in 66,303 ovarian nuclei/cells (29,920 nuclei from CG and 36,383 nuclei from control groups), capturing 988–2005 median number of genes per nuclei. Data from CG and control samples were combined for downstream analysis using Seurat-based workflow. To elucidate the cellular heterogeneity of ovarian stroma, we performed unsupervised clustering and annotated each cluster based on the canonical cell-type specific marker genes reported in single-cell human ovary atlas studies [26–28]. UMAP analysis showed multiple clusters which were combined to represent the seven distinct major cell categories of the human ovary with significantly enriched canonical marker genes for oocytes, granulosa, endothelial, smooth muscle, stromal, epithelial and immune cells (Fig. 1B and C).

UMAP plots showing the distribution of all the nuclei obtained from CG and control samples are shown in Fig. 1D. The two ovary specific cell types were identified as granulosa cells (*AMH*,* FSHR*,* FST* and *HSD17B1*) and oocytes (*DDX4*,* TUBB8*,* FIGLA* and *ZP3*) based on their signature marker genes (Fig. 1C, Figure S2A). Stromal cells which accounted for more than 50% of the total cell population (Fig. 1E, Figure S2B) expressed marker genes such as *PDGFRA*,* COL6A3*,* PTCH2*, and *STAR*. The least populated cells identified were the immune cells (Fig. 1E, Figure S2B) enriched with genes such as *PTPRC*,* CD163*,* IL10RA*, and *MS4A6A* (Fig. 1C). Other cell type clusters identified include endothelial cells (EC; *VWF*, *CD34*,* EGFL7* and *DIPK2B*), smooth muscle cells (*RGS5*, *LMOD1*,* MYOM1* and *MYH11*), epithelial cells (*FYB2*,* BNC1*,* PAX8*, and *ANXA13*; Fig. 1C, Figure S2A). Furthermore, GO analysis of differentially expressed marker genes identified across cell types revealed features corresponding to known biological functions and characteristics of each cell cluster, verifying the accuracy of cell clustering (Figure S2C). For instance, the genes with high expression in granulosa cells were enriched in GO terms such as steroid hormone biosynthesis and activin receptor signaling. GO terms specific to the immune cluster included regulation of cytokine production and antigen receptor-mediated signaling. GO terms including blood vessel morphogenesis and sprouting angiogenesis were enriched in endothelial cell cluster. (Figure S2C).

To spatially map ovarian cell types defined by our snRNA-seq analysis, we performed Visium ST analysis on hematoxylin eosin-stained ovary sections (Figure S3A) from CG and control patients, which generated whole-transcriptome data and retained spatial information. Following data integration, clustering analysis and cell-type annotation based on canonical marker genes from snRNA-seq data, we spatially mapped seven major ovarian cell types in the ST (Fig. 1F). The ST mapping of human ovaries illustrates the spatial distribution of these seven different cell types highlighting the organization of the ovarian stroma and the presence of distinct follicles (oocytes and granulosa cells) at the cortex region (Fig. 1F; Figure S3B-D). Other identifiable cell types, including endothelial cells, immune cells, epithelial cells and smooth muscle cells were found within the ovarian stroma of CG and control group (Figure S3B-D). Spatial distribution of ovarian cell types was depicted as individual spatial feature plots showing spot-based enrichment of each cell type (Figure S4).

### Dynamic gene expression patterns and altered signaling pathways in immune cell cluster

**Fig. 2:**
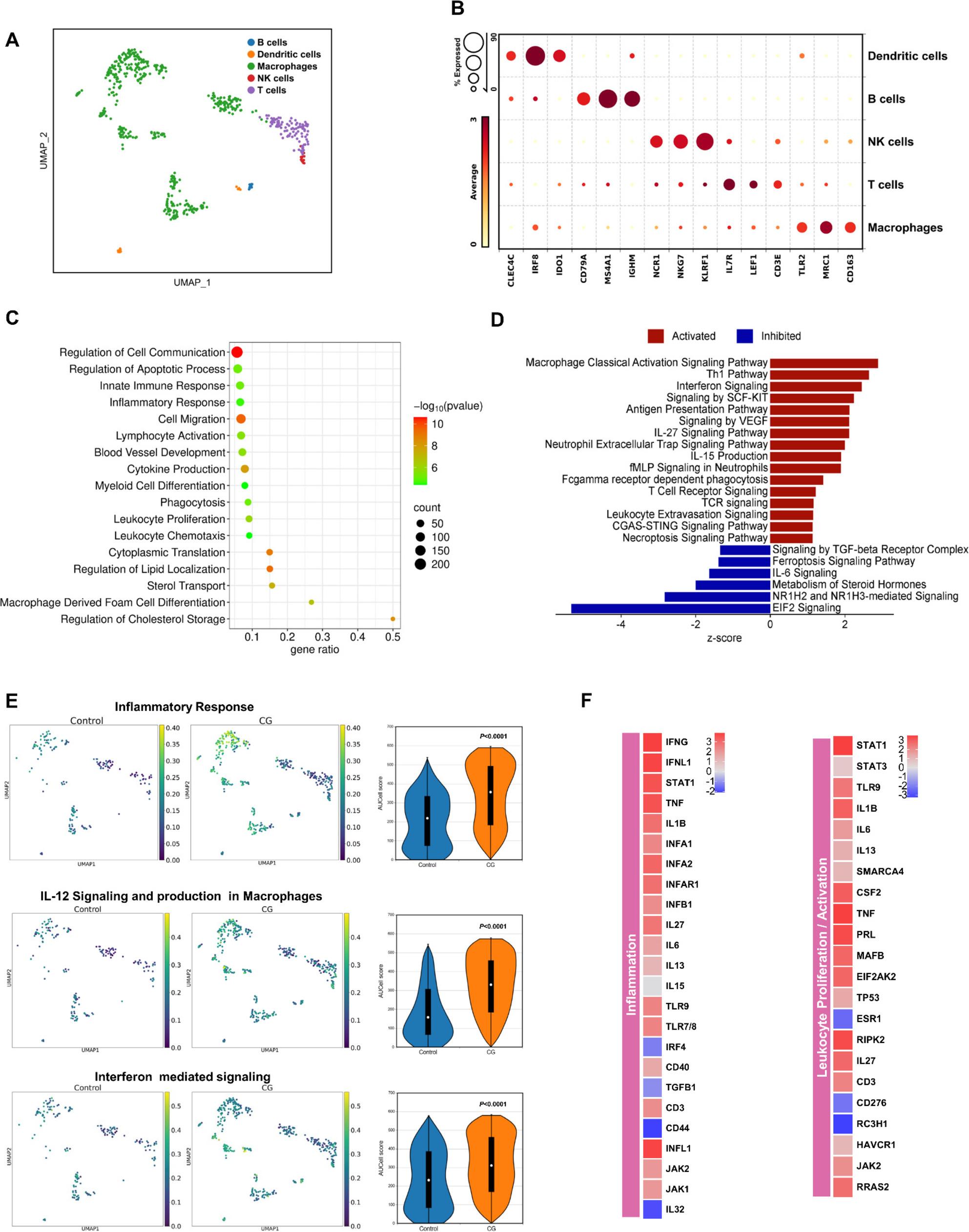
Transcriptomic profiles and altered signaling pathways in immune cell cluster. **A** UMAP plot showing 5 different immune cell types identified in human ovarian transcriptomic atlas. **B** Bubble plot of representative marker genes for annotating the immune cell populations. **C** GO enrichment plot showing various biological processes associated with DEGs that were significantly enriched in the immune cluster. **D** IPA analysis on the DEGs from immune cells displaying activated/upregulated and inhibited/downregulated pathways in the CG group. **E** UMAP visualization (left) and violin (right) plots showing the differences in the inflammatory response, IL-12 signaling in macrophages and interferon signaling pathway AUCell score between immune cells of CG and control. Unpaired t-test was used to compare AUCell scores between CG and control groups showing statistically significant *P* values. **F** IPA upstream regulator analysis of immune cluster DEGs showing activated/inhibited upstream genes (CG vs. control, z-score) that are associated with inflammation and leukocyte proliferation/activation

We re-clustered the single-cell profiles corresponding to the immune cluster, which resulted in identification of 5 different populations of immune cells: B cells, dendritic cells, macrophages, NK cells and T-cells expressing canonical signature marker genes (Fig. 2A and B). Macrophages are the abundant immune cell type within the ovary, and their proportion is much higher in the CG group compared to the control group (Figure S5A and B). We conducted a comparative analysis of gene expression patterns between CG and control samples in an immune cell cluster and performed gene enrichment analysis on differentially expressed genes (DEGs). GO enrichment analysis revealed that biological processes associated with innate immune response, inflammatory response, cell migration/communication, cytokine production, leukocyte proliferation/chemotaxis, myeloid cell differentiation and regulation of cholesterol storage were significantly enriched (Fig. 2C). IPA enrichment analysis revealed that many important pathways related to immune response/regulation were significantly activated/upregulated such as macrophage activation signaling, Th1 signaling, interferon signaling, antigen presentation pathway, IL-27 signaling, FCGR-mediated phagocytosis, T cell receptor signaling, leukocyte extravasation signaling and more (Fig. 2D). In contrast, several genes associated with pathways such as NR1H2 and NR1H3-mediated signaling, IL-6 signaling, ferroptosis pathway and TGF-β signaling were downregulated (Fig. 2D). We investigated the expression of a few inflammation-related pathways in the immune cluster at the single-cell level by calculating the AUCell scores for pathways based on the expression of the signature genes (annotated gene sets) using the AUCell package. We found pathways such as inflammatory response, IL-12 signaling & production in macrophages and interferon signaling were highly activated in CG compared to controls (Fig. 2E). Furthermore, we performed upstream regulatory network analysis on DEGs using the IPA platform, to identify/predict upstream regulator genes that regulate the expression of their downstream target genes in our snRNA-seq dataset. This analysis revealed the upregulation of several upstream regulators associated with inflammation and leukocyte proliferation/migration (Fig. 2F).

### Spatial map of distinct ovarian immune cells and gene expression dynamics in macrophages

**Fig. 3:**
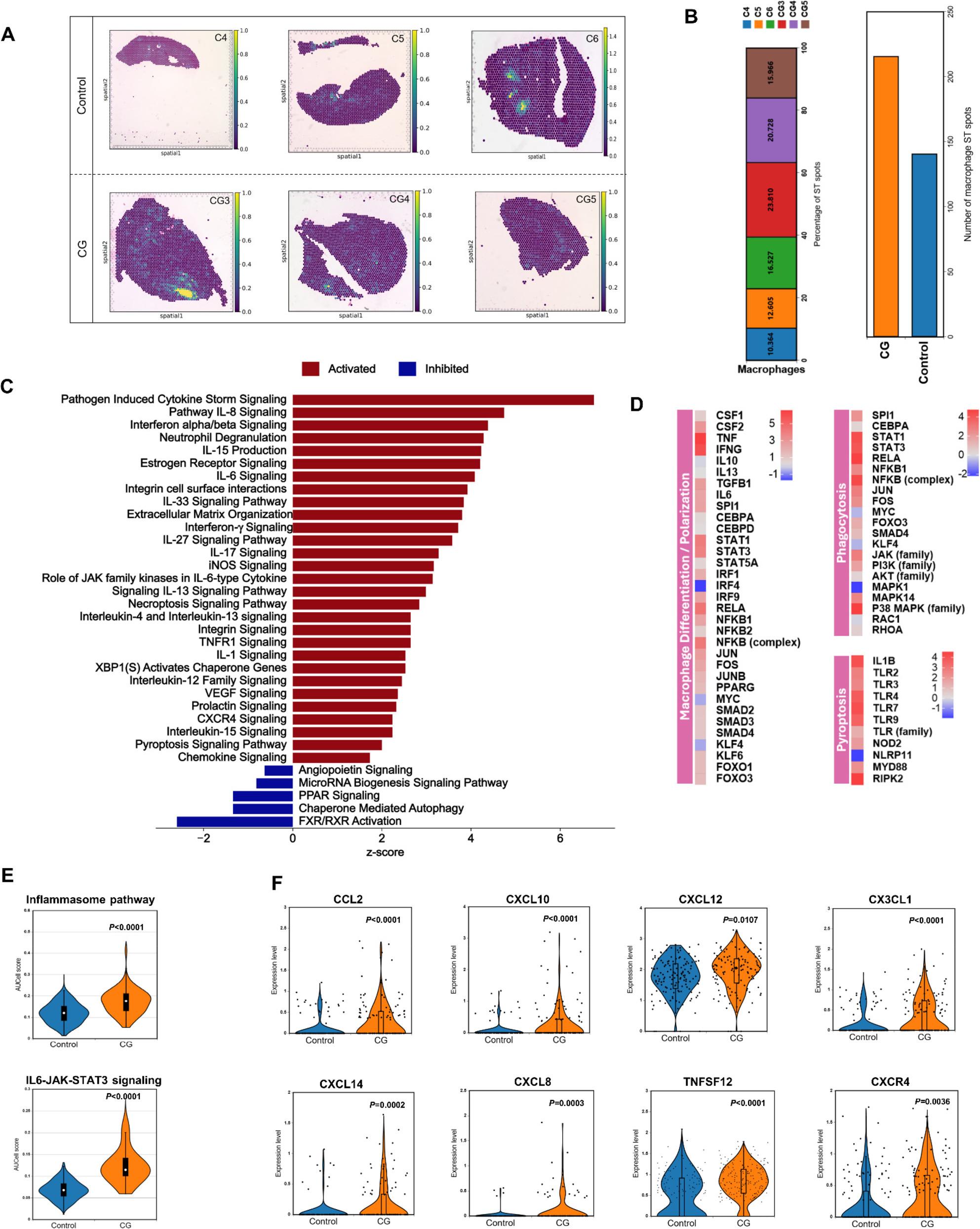
Spatial transcriptomic map of major ovarian immune cells and gene expression dynamics in macrophages. **A** Spatial feature plots displaying the spatial distribution of macrophages in the ovary sections of all the CG and control samples after snRNA-seq data integration. **B** Stacked bar plot showing percentage composition of macrophage ST spots among all the 6 ovary samples and bar graph showing number of macrophage ST spots between CG and control group. **C** IPA analysis of DEGs from macrophages displaying activated and inhibited signaling pathways in CG group. **D** IPA upstream regulator analysis of macrophage cluster DEGs showing list of activated/inhibited upstream genes (CG vs. control, z-score) that are associated with macrophage differentiation/polarization and macrophage mediated phagocytosis and pyroptosis. **E** Violin plots showing the differences in the inflammasome pathway and IL-6-JAK-STAT3 signaling pathway AUCell scores between macrophages of CG and control. **F** Violin plots displaying gene expression levels (CG vs. control) of important cytokines and their receptors involved in pro-inflammatory immune response. Unpaired t-test was used to compare AUCell scores and gene expression levels between CG and control groups showing statistically significant *P* values

Using the Cell2location deconvolution method, the spatial localization of immune cell types was determined in our six Visium ST samples. The reference gene expression signatures of five different immune cells (T/B/NK cells, dendritic cells and macrophages) from our snRNA-seq profiles were used to deconvolute the multi-cell ST map, thereby estimating the relative proportion of each cell type at each spatial location/spot (Figure S6A). We then spatially mapped the distribution of macrophages in CG and control ovary sections based on spot-based enrichment of canonical macrophage markers from snRNA-seq data (Fig. 3A). Our ST data clearly showed the presence of macrophage ST spots in the ovarian stroma of all six samples, and we noticed increased proportion of macrophage spots in the CG group compared to the control group (Fig. 3B). We then performed GO and IPA analyses on DEGs from macrophages to identify altered/enriched biological functions and signaling pathways. GO analysis revealed several genes involved in biological processes such as macrophage activation/migration, regulation of inflammatory response, response to cytokines, Fc-gamma receptor signaling, phagolysosome assembly and TNF-mediated signaling pathway profoundly enriched in CG (Figure S6B). IPA analysis showed that various cytokine signaling (IL-1, IL-6, IL-8, IL-13, IL-15, IL-17, IL-23 and Interferon), iNOS signaling, Integrin signaling, TNFR1 signaling, VEGF signaling, necroptosis signaling and pyroptosis signaling pathways were significantly activated/upregulated in the CG group (Fig. 3C). Moreover, upstream regulatory analysis revealed significant upregulation of several upstream regulator genes participating in inflammatory response, macrophage differentiation/polarization, macrophage mediated phagocytosis and pyroptotic cell-death (Fig. 3D; Figure S6C). Notably, we found that the inflammasome pathway and IL-6-JAK-STAT3 pathway activity score was higher in the macrophages of CG ovary (Fig. 3E). Explicitly, we found some key genes (*NLRP3*,* NFKB2*,* AIM2*,* TXNIP*,* PANX1*, and *GSDMD*; excluding *IL1B* and *CASP1*) associated with NLRP3 inflammasome pathway which play a crucial role in inflammation driven pyroptotic cell death was significantly upregulated in CG compared to control (Figure S6D). Additionally, specific pro-inflammatory mediators (*CCL2*,* CXCL8/10/12/14*,* CX3CL1*,* TNFSF12*,* and CXCR4)* involved in macrophage mediated inflammatory immune response in our spatial data were significantly upregulated in the CG group compared to control group (Fig. 3F).

### Characterization of transcriptionally distinct ovarian macrophage subsets

**Fig. 4:**
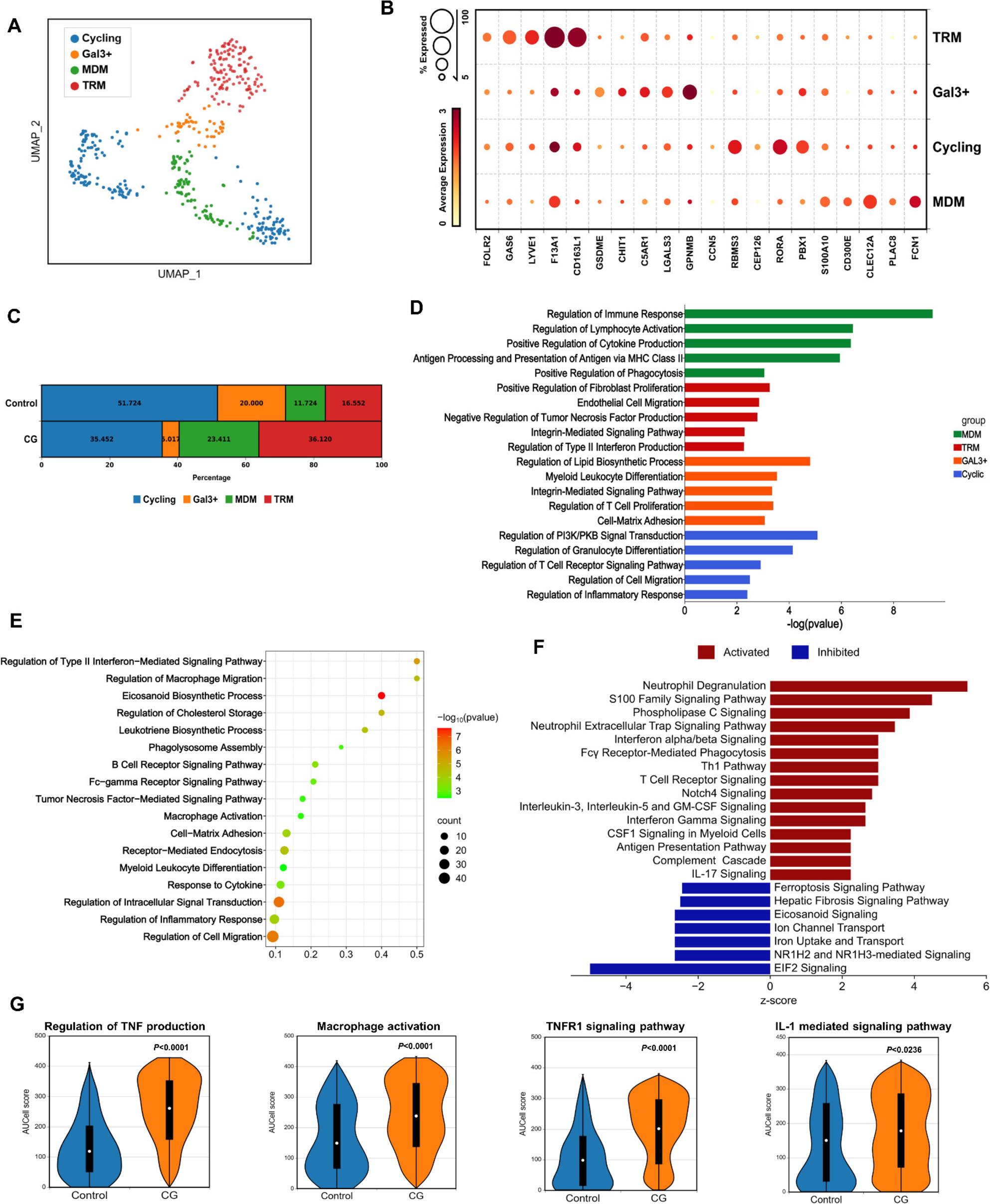
Identification and characterization of ovarian macrophage subtypes with transcriptomic diversity. **A** UMAP plot displaying 4 distinct macrophage subpopulations/subtypes identified in the human ovary atlas. **B** Bubble plot showing the distinct transcriptomic signatures (genes) identified in each macrophage subtypes from sub-clustering analysis. **C** Box plot represents the percentage composition of each macrophage subtype in CG and control groups. **D** GO enrichment plot showing various biological processes associated with the DEGs from 4 different ovarian macrophage subpopulations identified in the snRNA-seq data. **E** GO enrichment plot showing various biological processes associated with DEGs that were significantly enriched in macrophages of the CG group. **F** Bar plot shows significantly activated and inhibited signaling pathways in CG group using IPA analysis of DEGs from macrophage cluster. **G** Violin plots exhibiting the differences in AUCell activity score of biological processes “regulation of TNF production”, “macrophage activation” and IPA pathways “TNFR1 signaling”, “IL-1 mediated signaling” between CG and control groups. Unpaired t-test was used to compare AUCell scores between CG and control groups showing statistically significant *P* values

Macrophages contribute to ovarian tissue homeostasis by regulating inflammation, tissue repair and remodeling of the ECM in the ovarian stroma [20, 21, 24]. Previous studies have indicated that ovarian macrophages are composed of a mixed population of tissue-resident macrophages (TRMs), which derive from the extra‐embryonic yolk sac and fetal liver before birth, and monocyte‐derived macrophages (MDM), which derive from the bone marrow after birth [24]. In our study, we identified and characterized four distinct macrophage subsets that reside in the ovarian stroma exhibiting extensive transcriptional diversity (Fig. 4A and B).

We classified these 4 macrophage subpopulations into monocyte-derived macrophages (MDMs), tissue resident macrophages (TRMs), Galectin-3 positive macrophages (Gal3+) and cycling macrophages (Cycling). Ovarian MDM subpopulations showed unique signature genes such as *CD300E*, *CLEC12A*, *PLAC8*, and *FCN1* (Fig. 4B). On the other hand, TRMs were characterized by increased expression of *FOLR2*, *LYVE1*, *GAS6*, and *CD163L* in our snRNA-seq dataset (Fig. 4B). Furthermore, MDMs displayed relatively higher levels of *CD52*,* CD86*,* CCR2*,* SELL*,* LYZ*, and *S100A8* (Figure S7A) compared to other subsets that resemble inflammatory macrophages previously described in human ovary and skeletal muscle single-cell atlases [28, 31]. TRMs showed higher expression of *C1QA CD36*,* A2M*, and *GAS6* (Figure S7A) which are associated with the classical complement pathway involved in clearance of apoptotic cells via phagocytosis, and regulation of T cell responses. “Gal3+”, another identified macrophage subset, showed specific expression of genes *LGALS3*,* LGALS1*,* CHIT1*, and *GPNMB*, which are implicated in macrophage-mediated ECM remodeling and fibrosis in various diseases (Fig. 4B). Finally, the “Cycling” macrophage subset expressed genes (*CCN5*,* CEP126*,* RBMS3* and *PBX1*) associated with chromatin, nucleosome, and cell cycle regulation (Fig. 4B). We noted higher cell proportion of MDMs and TRMs in the CG group when compared with the control group (Fig. 4C). In contrast, the percentages of Gal3+ and cyclic macrophages were higher in control compared to CG ovaries (Fig. 4C).

GO analysis showed that the marker genes in the MDM subcluster were mainly enriched in GO terms such as regulation of lymphocyte activation, positive regulation of cytokine production, and antigen processing and presentation, suggesting their role in pro-inflammatory immune response (Fig. 4D). However, TRMs were enriched in biological processes mainly related to negative regulation of TNF production, fibroblast proliferation and phagocytosis, supporting the previously described notion that TRMs play a critical role in maintaining tissue homeostasis by promoting tissue repair, clearing out cell debris and resolving inflammation (Fig. 4D). Single-cell cytokine profiling using AUCell analysis was performed to find out heterogeneity in the subsets of macrophages with respect to cytokine signaling. We found activity score of several of the cytokines signaling (TNF, IFN, IL-1/6/12/17/18 and CXCL12) that are known to induce pro-inflammatory response is relatively higher in MDMs compared to other macrophage subsets (Figure S7B).

To further explore the biological functional pattern of the immune-associated genes differentially expressed in macrophages of CG and control groups, we performed GO and IPA enrichment analyses. We found that several genes predominantly enriched in GO functional terms were immune-related genes such as regulation of type-II interferon-mediated signaling, macrophage activation/migration, TNF-mediated signaling, cell-matrix adhesion, response to cytokines and regulation of inflammatory response (Fig. 4E). The IPA enrichment analysis showed that several genes participating in neutrophil degranulation, S100 family signaling, FCGR-mediated phagocytosis, Interferon signaling, Th1 pathway and IL-3/IL-5/GM-CSF/IL-17 signaling were activated in the CG group (Fig. 4F). In contrast, we found that EIF2 signaling, NR1H2/NR1H3-mediated signaling, fibrosis signaling and ferroptosis signaling pathways were downregulated in the CG ovary (Fig. 4F). Furthermore, we found that “Regulation of TNF production” and “Macrophage activation” biological processes, and “TNFR1 signaling” and “IL-1 mediated signaling” pathways AUCell scores were higher in the CG group compared to the control group (Fig. 4G).

### MDMs enriched in the ovary of CG trigger inflammatory immune response mediated by cytokines

**Fig. 5:**
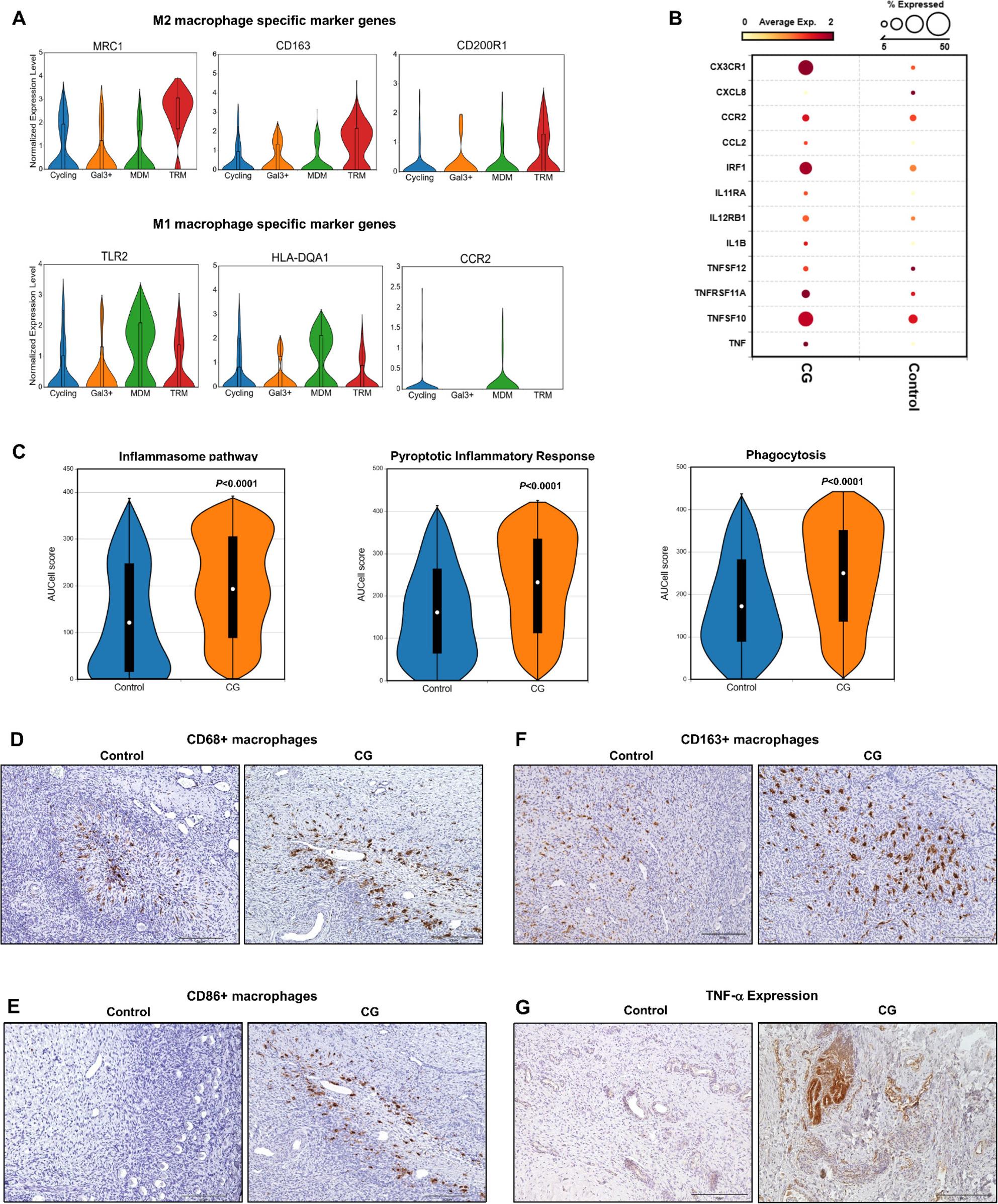
Distinct phenotypes of MDMs and TRMs mediating inflammatory immune response in CG ovary. **A** Violon plots showing expression of specific markers genes unique to M1 and M2-like macrophage phenotypes in four macrophage subtypes. **B** Bubble plot showing gene expression of pro-inflammatory mediators/cytokines between CG and control in the MDMs. **C** Violin plot showing the AUCell activity score of NLRP3 inflammasome pathway in macrophages between CG and control groups. Unpaired t-test compared the AUCell scores between CG and control group showing significance *P* < 0.001. IHC images show localization of CD68+ (**D**), CD86+ (**E**), CD163+ (**F**) macrophages and TNF-α expression (**G**) in the ovary sections of CG and control patients (n = 3)

Macrophages are mainly classified into pro-inflammatory and anti-inflammatory states which are more commonly defined as M1 and M2 subtypes, respectively [32, 33]. To characterize the inflammatory states of the ovarian macrophages, we compared all four macrophage subsets to well-defined M1 and M2 polarization/activation signatures previously reported [32–34]. Interestingly, we found that ovarian MDMs expressed higher levels of marker genes (*TLR2*,* HLA-DQA1*,* CCR2*,* IL1B*,* IL12B*,* STAT1*,* CCL4 and CXCL8*) that are associated withpro-inflammatory M1-like macrophage phenotype (Fig. 5A; Figure S7C). While TRMs behaved more like the M2 phenotype with enriched genes (*MRC1*,* CD163*,* CD200R1*,* CD209*,* IL10*,* A2M*,* TGFB3*, *COLEC12* and IGF1) that play a crucial role in resolution of inflammation, clearance of cell debris and tissue repair (Fig. 5A; Figure S7C) [28, 33, 34]. We also compared the expression of few inflammatory genes and cytokines between the CG and control groups in the MDM cluster. We found that cytokines such as *TNF*,* TNFSF10*,* IL-1B*, and *CCL2* which are proinflammatory in nature and cytokine receptors such as *CX3CR1*,* CCR2*,* IL11RA*, and *IL12RA* were highly expressed in CG compared to control groups (Fig. 5B). Meanwhile, using AUCell analysis, we scored the activity of NLRP3 inflammasome pathway that triggers pyroptosis, which showed increased inflammasome activity in MDMs compared to TRMs. Further we noticed key genes involved in the NLRP3 inflammasome mediated pyroptotic cell death are highly expressed in MDMs compared to TRMs suggesting that MDM subpopulation is pyroptotic in nature likely contributing to proinflammatory response (Figure S7D and E). The AUCell activity scores of “Inflammasome pathway”, “pyroptotic inflammatory response” and “phagocytosis” in macrophages were higher in CG compared to control (Fig. 5C). Additionally, we performed IHC analysis on ovary sections from CG and control groups of patients to localize and compare the distribution of these two macrophage phenotypes TRMs and MDMs using antibodies against surface CD163 and CD86 makers, respectively [33–35]. CD68+ staining revealed the overall macrophage population was moderately higher in the CG group compared to the control group (Fig. 5D). More specifically, we found a very profound increase of MDMs in the CG ovary when compared to that in the control ovary, evident from CD86+ staining suggesting presence of clusters of activated macrophages in the ovarian stroma of CG, while it was hardly noticed in controls (Fig. 5E). The CD163+ staining detected the presence of relatively larger clusters of TRMs inside the stroma of the CG ovaries when compared to controls (Fig. 5F). These results suggest that there is increased population of activated macrophages, specifically the pro-inflammatory MDM subsets in the CG ovary, which also correlates with the snRNA-seq data. We also found increased expression of NLRP3 and CASP-1 in the primordial follicles and stromal cells of CG ovary when compared to control ovary, suggesting NLRP3 mediated inflammasome activation (Figure S7F and G). Furthermore, IHC analysis showed that the expression of the key pro-inflammatory cytokine TNF-α, which plays a significant role in triggering and amplifying inflammatory response was much higher in the ovarian stroma and in the blood vessels of CG when compared to control group (Fig. 5G).

### Diverse transcriptional signatures in ovarian vascular and lymphatic endothelial cells modulating immune cell trafficking

**Fig. 6:**
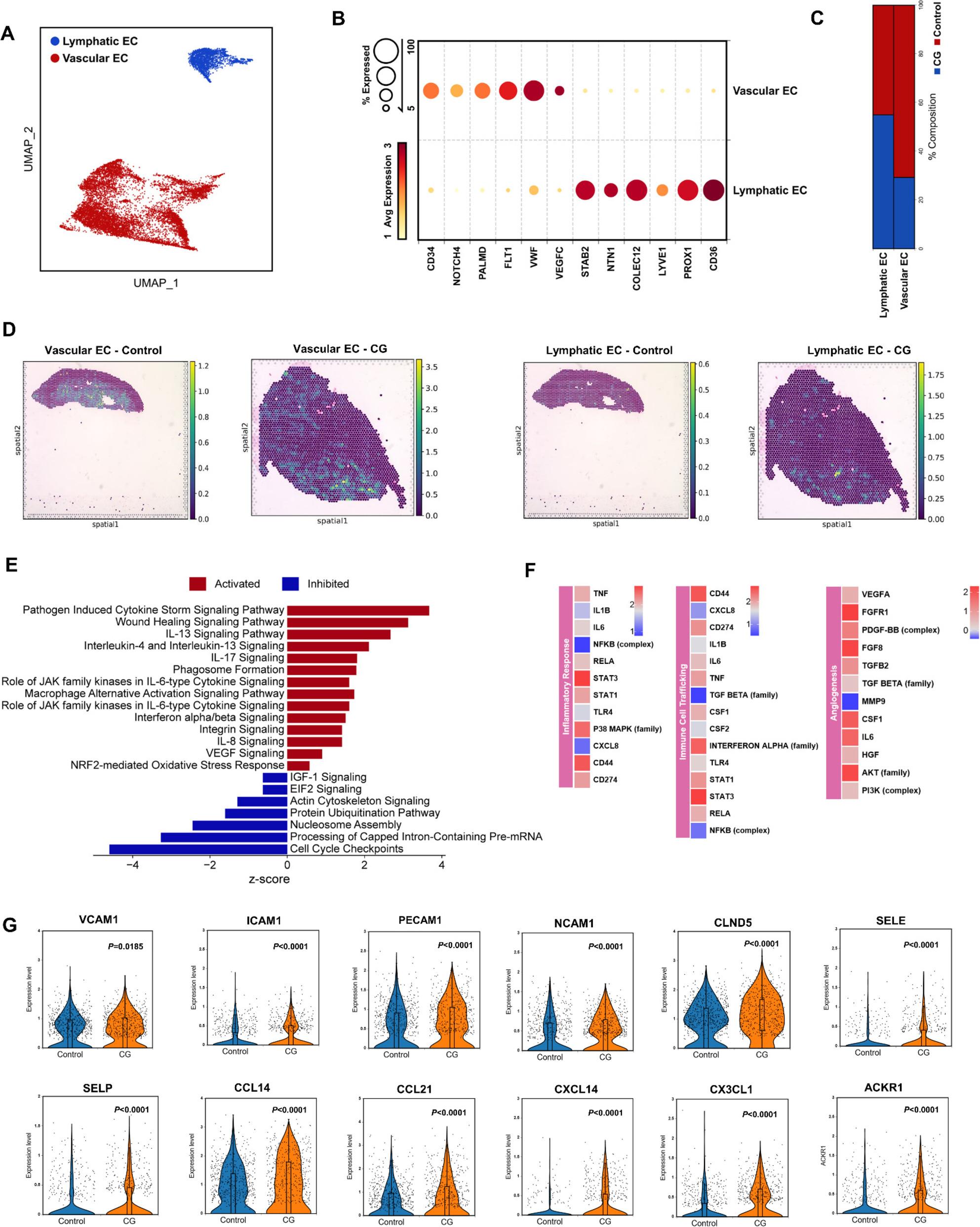
Single nucleus and spatial transcriptomic profiles of ovarian endothelial cells and their role in immune modulation. **A** UMAP plot showing two distinct subclusters of ECs: vascular and lymphatic ECs identified in the human ovary atlas. **B** Bubble plot showing the distinct transcriptomic signatures (marker genes) identified in each vascular and lymphatic EC subclusters. **C** Box plot represents the percentage composition of two ECs subpopulations in CG and control ovaries. **D** Representative spatial feature plots (CG and control) showing identification/mapping of vascular and lymphatic EC enriched ST spots based on snRNAseq reference marker genes. **E** IPA analysis on DEGs (CG vs. control) from ECs showing activated and inhibited signaling pathways in CG group. **F** IPA upstream regulator analysis on DEGs showing activated/inhibited upstream genes (CG vs. control, z-score) that are associated with inflammatory response, immune cell trafficking and angiogenesis. **G** Violon plots exhibiting expression levels of key cell adhesion genes and chemokines that participate in EC mediated extravasation/diapedesis. Unpaired t-test was used to compare the gene expression levels between CG and control groups showing statistically significant *P* values

ECs play a crucial role in immunomodulation by regulating immune cell trafficking, activation status and function. In our study, we characterized ECs based on transcriptomic signatures to understand gene expression dynamics in CG ovary that may influence recruitment of immune cells and inflammatory response. In our initial cell clustering analysis, we obtained two main distinct clusters of endothelial cells: “Vascular EC” and “Lymphatic EC” (Fig. 6A). The differential gene expression analysis between these two endothelial clusters revealed unique transcriptomic signatures pertaining to vascular and lymphatic ECs with distinct biological functions (Fig. 6B). GO and Reactome pathway enrichment analysis showed that the marker genes in both EC subclusters were mainly enriched in blood vessel morphogenesis, VEGF signaling and regulation platelet aggregation (Figure S8A). The marker genes in vascular ECs were mainly enriched in granulocyte differentiation, cell migration, platelet degranulation and NOTCH signaling. However, lymphatic EC marker genes were primarily involved in biological processes/pathways related to lymphatic EC differentiation, regulation of cell-matrix adhesion, regulation of lymphocyte, antigen processing-cross presentation and chemokine signaling (Figure S8A). We found that the lymphatic EC population was much higher in CG ovaries when compared to controls (Fig. 6C).

Integrative analysis using cell2location method enabled us to spatially identify vascular and lymphatic subtypes of ECs in the Visium ST data from ovary sections. The spatial expression of signature marker genes unique to the two EC subtypes is shown in the spatial feature maps (Fig. 6D; Figure S8B). We then performed IPA pathway enrichment analysis on the DEGs to explore the biological functional pattern relevant to immune modulation. IPA pathway analysis revealed upregulation of several pathways such as pathogen induced cytokine storm, S100 family, wound healing, cGAS-STING, eNOS, VEGF, IL-6/8/13/33 signaling, and other biological functions such as ECM organization and Integrin cell surface interactions (Fig. 6E). Furthermore, upstream regulatory analysis revealed most of the upstream regulator genes regulating inflammation, immune cell recruitment and angiogenesis processes were activated in CG group (Fig. 6F). More interestingly, we also discovered that gene expressions of cell-adhesion molecules (*VCAM1*,* ICAM1*,* PECAM*,* NCAM1*,* CLND5*,* SELE*, and *SELP*) and chemokines/chemokine receptors (*CCL14*,* CCL21*,* CXCL14*,* CX3CL1*, and *ACKR1*) that play a crucial role in immune cell extravasation and recruitment into surrounding stromal tissue at the site of inflammation were significantly upregulated in the endothelial cells of CG when compared to control ovaries (Fig. 6G). Other immunomodulatory genes (*IL-33*,* TNFSF12*,* CD44*,* JAM2*,* NRP2*,* ITGA5/8*,* ITGAV*,* SDC3*, and *MMP17*) participating in downstream signaling that regulate/influence endothelial cell behavior, vascular permeability and transendothelial migration of immune cells were also significantly changed (Figure S8C).

### Cell-cell communication analysis using CellChat reveals dramatic changes in ovarian interactome

**Fig. 7:**
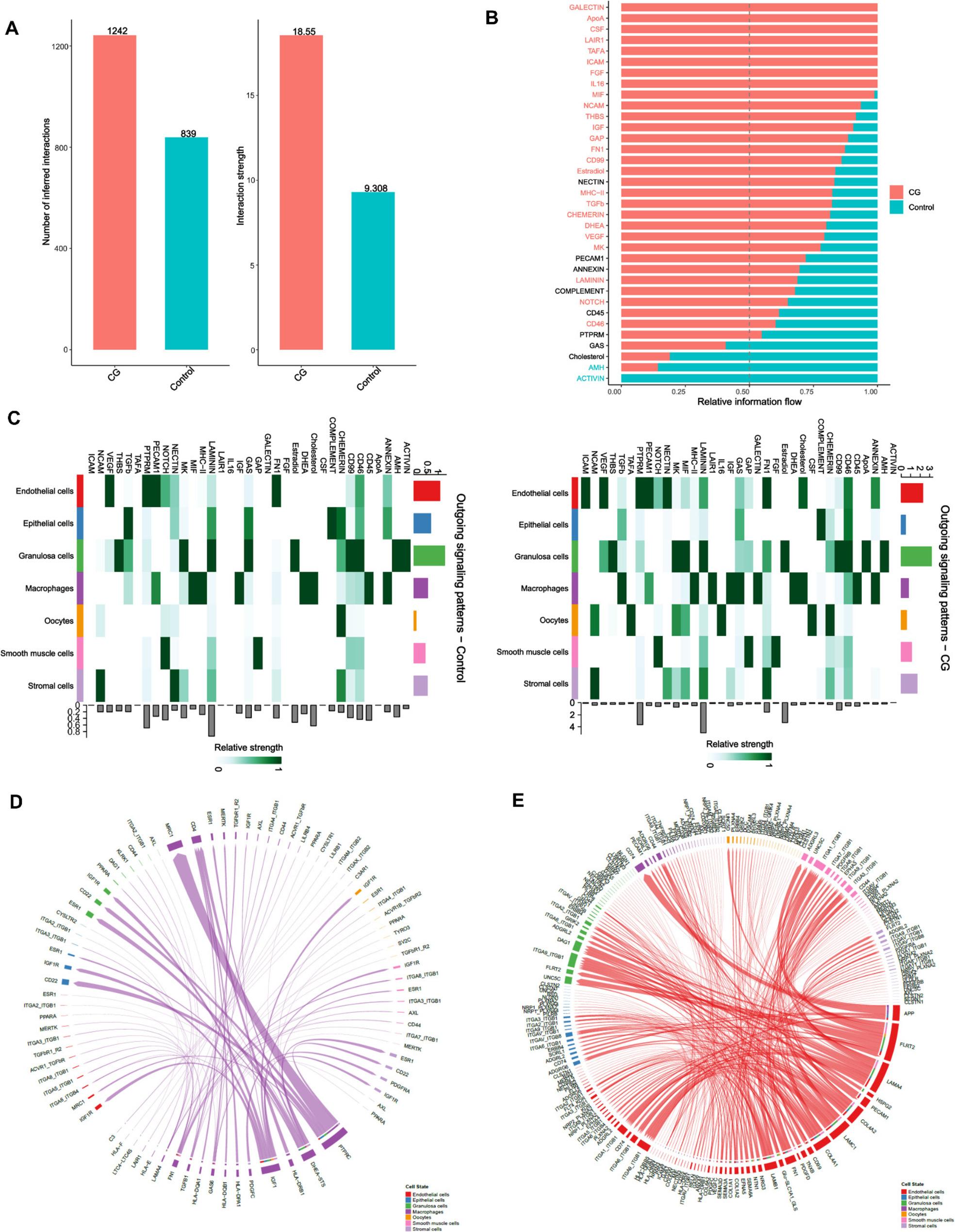
Intercellular communication network among different ovarian cells. **A** Bar plot showing total number of interactions and strength/weight of interactions in CG and control samples. **B** Bar plot showing the comparison of different signaling pathways based on the relative information flow between control and CG samples. **C** Heatmap showing changes in outgoing signaling patterns for each signaling pathway in each cell population between two conditions. The bottom row bar specifies the sum of outgoing signaling strength across all cell groups for one pathway. The right row bar height shows the sum of outgoing signaling strength across all pathways for one cell group, which is much stronger in CG compared to control group. **D **Chord diagram illustrates all the ligand-receptor pairs and their weight contributing to the activated signaling from macrophages to the other signaling receiving/target cells. **E** Chord diagram showing all activated signaling pathways along with their ligand-receptor pairs from ECs to the other signaling receiving cells

We performed cell-cell communication analysis to gain further insights into the intercellular interactions between macrophages and other cell types in the ovarian microenvironment. The CellChat tool was used to quantitatively infer and analyze intercellular communication networks from scRNA-seq data, where we first compared the overall probability of the number of ligand-receptor pair interactions among different cell types in both groups. We found more numbers of cell-cell interactions and an increase in interaction strength in the CG group when compared to the control group (Fig. 7A). The strength of interactions was stronger among endothelial cells, oocytes, granulosa cells, stromal cells and smooth muscle cells indicating a high probability of cellular communications within these cell types in CG ovary (Figure S9A). We also examined the differential outgoing and incoming interaction strengths associated with each cell type. We found that both outgoing and incoming signaling of oocytes, endothelial, smooth muscle and granulosa cells were enhanced in the CG when compared with the control group (Figure S9B). Compared with the control group, the incoming signals of macrophages and outgoing signals of oocytes and stromal/thecal cells were also strengthened in the CG group (Figure S9B). In addition, the ligand-receptor pairs detected in the cell types were categorized into several signaling pathways, where we compared the information flow of each signaling pathway between the two groups. We found that compared with controls more than half of the signaling pathways were highly enriched/enhanced in the CG group (Fig. 7B).

Some of them are immune related signaling pathways: GALECTIN, PECAM1, CSF, IL-16, MIF, CD99, and MHCII that play an important role in inflammatory immune response (Fig. 7B). Interestingly, we noted that AMH and ACTIVIN signaling pathways, which are essential for ovarian follicle growth and development, were enhanced in the control group (Fig. 7B). To further analyze the alteration of signals in each cell population, we compared the signal strength of each signaling pathway between CG and control groups. We found that several outgoing/incoming signaling pathways involved in the regulation of the immune system such as GALECTIN, CSF, LAIR1, CD46, IL-16, MIF, FN1, and CD99 were specifically enriched in the macrophages of CG samples. ICAM1, ANNEXIN, CD46, FN1, GAS, NECTIN, PECAM1, and LAMININ outgoing signaling strength were enhanced in the endothelial cells of the CG group (Fig. 7C; Figure S10). We also noticed that in granulosa cells the outgoing signaling strength across most of the pathways was much higher in CG samples (Fig. 7C). Additionally, we studied the ligand-receptor pairs in the active/upregulated signaling pathways in CG and found that macrophages and ECs interact with their main receivers (other ovarian cells) via various paracrine signaling (Fig. 7D and E). Using circle plots, we further compared (CG vs. control) changes in the number/strength of specific signaling pathways coming from macrophages and endothelial cells. For instance, we noticed more MIF and CD99 signaling interactions between macrophages and other cell types in the CG group (Figure S11). CD45 and MHC-II signaling strength increased within the macrophages of the CG group compared to the control group (Figure S11 and 12). Compared with controls, we observed increased FN1, PECAM1, LAMININ, CHEMERIN, and ANNEXIN signals that are involved in cell migration and adhesion from ECs to macrophages in the CG group (Figures S12 and S13).

### D-galactose toxicity causes oxidative stress induced cascade of inflammatory response mediated by NLRP3 inflammasome pathway

**Fig. 8:**
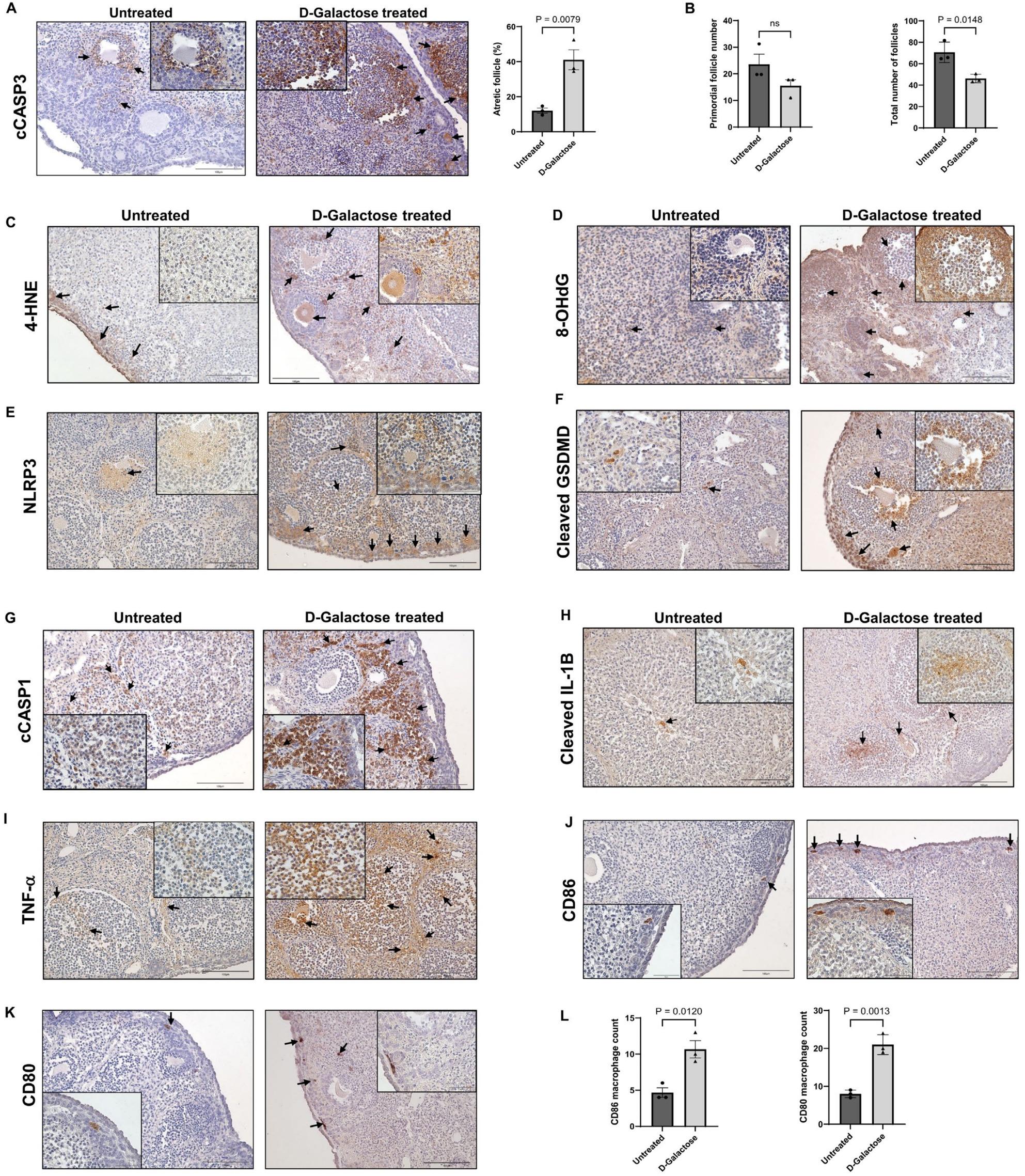
D-galactose toxicity induces oxidative stress triggering NLRP3 inflammasome mediated pyroptotic cell death in mouse ovary. **A**) Representative IHC stained images showing expression of cleaved-CASP3 indicating follicular atresia, and a bar graph showing the percentage of atretic follicles in the D-galactose treated and untreated mouse ovary sections (*n*=3). **B**) Bar graphs showing the primordial follicle number and total number of follicles in the D-galactose treated and untreated mouse ovaries. Representative IHC stained images showing expression of 4-HNE (**C**), 8-OHdG (**D**), NLRP3 (**E**), cleaved GSDMD (**F**), cleaved CASP-1 (**G**), cleaved IL-B (**H**), TNF-alpha (**I**), CD86 (**J**) and CD80 (**K**) in the D-galactose treated and untreated mouse ovaries (n=3). L) Bar graphs showing the CD86 and CD80 positive macrophage count in the D-galactose treated and untreated mouse ovaries. Unpaired t-test was used to compare CG and control groups showing statistically significant P values. Arrows indicate IHC DAB signal. Scale bar indicates 100 um. Higher magnification (60x oil immersion lens) images are shown as insets within the main IHC image

In mice, D-galactose and its metabolites were shown to induce oxidative stress via increased ROS production leading to apoptosis of granulosa cells and poor quality of oocytes [13, 14]. Previously, we have shown that D‐galactose toxicity causes increased follicular atresia/cell-death in mouse whole ovary cultures [16]. Here, we treated mouse ovary cultures with 50mM of D-galactose to evaluate the role of oxidative stress in the activation of inflammasome pathway that can trigger inflammation and pyroptotic cell death. After D-galactose treatments, we assessed the impact on the ovarian follicles by counting the primordial follicles, total follicle number, and calculating the percentage of atretic follicles (cleaved CASP-3 expression). We performed IHC analysis on ovary sections to check the expression of 8-OHdG and 4-HNE (indicator of oxidative stress), NLRP3, cleaved GSDMD, cleaved CASP1 and cleaved IL-1β (activation of inflammasome pathway and pyroptosis), TNF-α (inflammation) and cleaved-CASP3 (follicular atresia). We found a significant increase in the percentage of atretic follicle (evident from cleaved CASP-3 expression in the granulosa cells) in the D‐galactose treated ovaries when compared to that in untreated ovaries, suggesting that galactose toxicity induces increased follicular atresia (Fig. 8A). Although there was no significant change in the primordial follicle number, the total follicle number was moderately reduced in the D‐galactose treated group (Fig. 8B). Expression of both oxidative stress markers 8-OHdG and 4-HNE were much higher in the ovarian follicles and stromal tissue of D‐galactose treated ovaries when compared with untreated ovaries indicating increased oxidative stress (Fig. 8C and D). We noticed a dramatic increase in expression of NLRP3, cleaved CASP-1, cleaved GSDMD and activated IL-1β in the D‐galactose treated group compared to the untreated group (Fig. 8E-H). Finally, higher expression of TNF-α expression was noticed in the follicles and stromal cells of D‐galactose treated ovaries compared to untreated ovaries (Fig. 8I). Taken together, our IHC data suggest that D‐galactose toxicity can induce oxidative stress, which can trigger activation of the NLRP3 inflammasome pathway contributing to inflammation, pyroptotic cell-death, and ovarian tissue damage. To find out whether D-galactose treatments have an impact on MDM population, we used CD86/CD80 markers to detect these activated macrophages in the ovaries of D-galactose and untreated mice. We found an increase in the MDM population inside the stroma of the D-galactose treated group compared to the untreated group (Fig. 8J-L). 

## Discussion

**Fig. 9:**
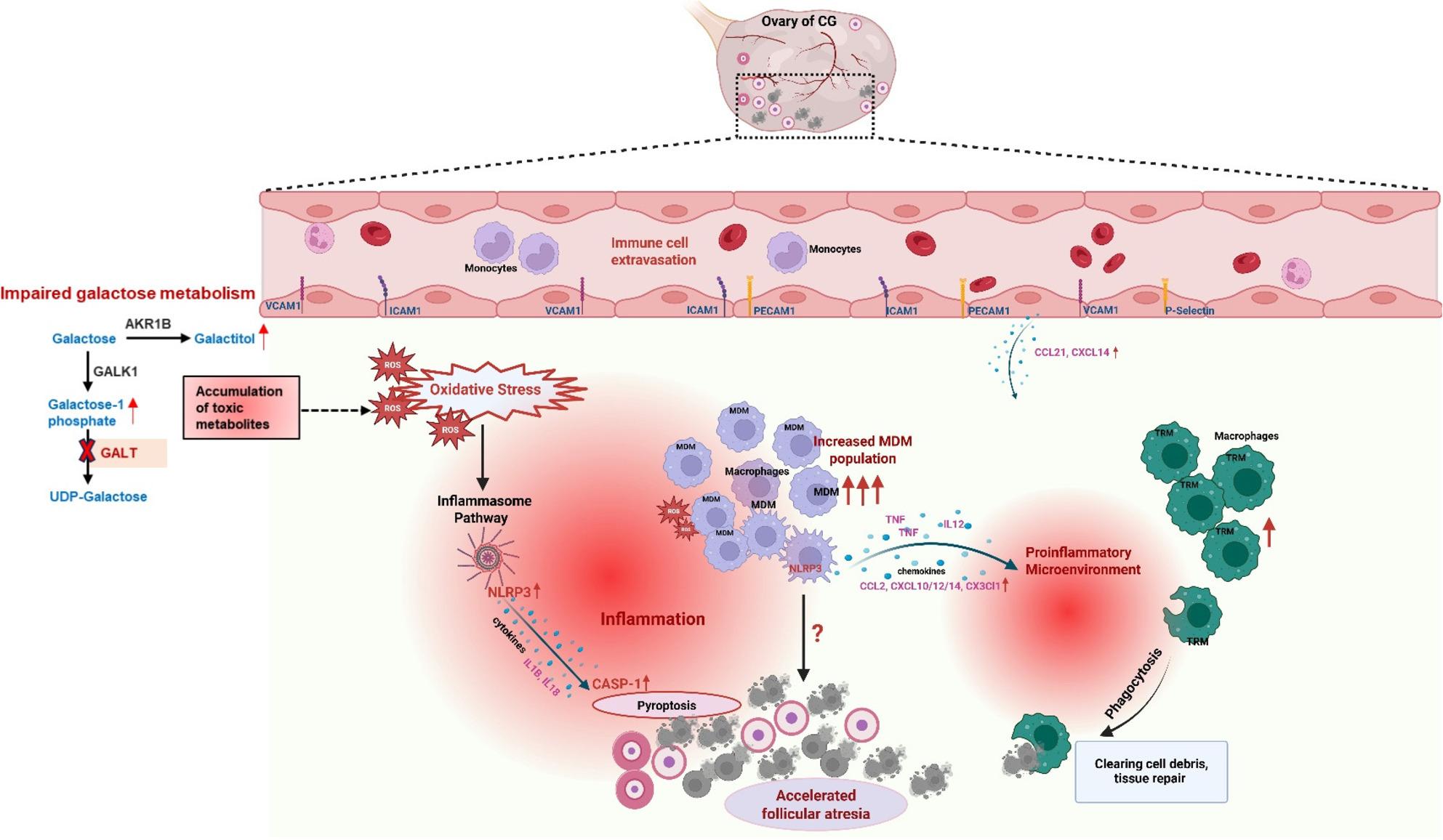
Proposed mechanism: Impaired galactose metabolism in CG causes accumulation of toxic metabolites such as galactitol and galactose-1-phosphate, which results in increased oxidative stress via increased ROS production in the ovary. This oxidative stress likely initiates a cascade of events involving activation of NLRP3 inflammasome which triggers CASP-1 mediated pyroptotic cell-death of ovarian follicles, contributing to pro-inflammatory microenvironment inside the ovarian stroma as evident from increased expression of TNF-α and IL-1β expression. Further, the proinflammatory microenvironment due to inflammasome pathway in the ovary is likely contributing to increased population of macrophages (MDMs and TRMs) at the site of inflammation, which is evident from our IHC data. We believe that all these events leading to aggravated inflammation negatively impact the follicles and ovarian function, contributing to early onset of POI condition in CG. However, the direct role of these activated macrophages in the follicle damage in CG ovary remains to be elucidated. Fig. 9 schematic diagram was created using BioRender software

CG is a rare metabolic disorder in children often associated with POI in females and may provide a unique model to evaluate the plausible role of the immune system in the onset of POI in prepubertal girls. In the present study, we took advantage of high-throughput snRNA-seq and Visium ST to generate a comprehensive transcriptomic landscape of the human ovary ovarian from prepubertal girls diagnosed with CG disorder and POI. We comprehensively profiled the transcriptional immune landscape of ovarian samples from CG and control patients, which allowed us to explore the complex interplay of key regulatory pathways and cellular interactions that can explain the advent of events affecting ovarian function in individuals with CG. Our snRNA-seq analysis of these ovaries revealed five different immune cell populations based on canonical marker genes, with macrophages as the predominant cell type. We successfully mapped the spatial location of immune cells and ECs in the ST data based on snRNA-seq reference marker genes using the Cell2location deconvolution method. In our transcriptomic analysis, we mainly explored changes in the gene expression profile of macrophages and ECs at single-cell resolution to broadly unravel the inflammatory immune responses that may adversely affect the ovarian follicle growth in CG. Subsequently, we also evaluated the cellular communications among the different major cell types by examining receptor-ligand–based interactions to better understand the signaling pathways regulating the immune response. Furthermore, we evaluated the effects of D-galactose toxicity on whole ovary tissue culture with respect to oxidative stress, activation of NLRP3 inflammasome and cell death of ovarian follicles (Fig. 9).

The immune system plays a crucial role in regulating ovarian function by influencing various stages of follicular development, ovulation, and corpus luteum formation, thereby ensuring the proper selection and maturation of follicles [17–19]. Studies have shown that macrophages, which predominantly express CD68 are the most abundant immune cell population in the human ovary, and are widely distributed in the theca, stroma, and corpus luteum regions [20–24]. Macrophages are multifaceted cells that play a critical role in both initiating and resolving inflammation. They secrete various types of cytokines and chemokines that direct inflammatory responses and aid in the tissue repairing process [32–34]. In mice, significant changes in macrophage polarization and activation occur during the postnatal development, with key shifts occurring around 6–8 weeks as they reach adulthood and establish distinct, tissue-specific resident macrophage populations. While many resident macrophages are established from embryonic precursors (yolk sac) early on, this period marks a critical stabilization of populations that, by adulthood, are essential for tissue homeostasis, vascular remodeling, and lipid metabolism [36]. Macrophage diversity is regulated by the combination of tissue-specific cues and inflammatory signals that drive distinct transcriptional programs. Single-cell transcriptomics have previously reflected this level of macrophage heterogeneity in a variety of organs [37, 38].

In our transcriptomic analysis, we found four distinct macrophage subpopulations exhibiting unique transcriptome signatures. Among these, MDMs and TRMs exhibited distinct phenotypes. It is well known that macrophages exhibit a considerable degree of plasticity defined by the two functional subsets: M1 (classically activated macrophages) and M2 (alternatively activated macrophages). M1 macrophages are defined by their expression of MHC Class II, CD80/CD86, iNOS and TLR2 [32–35]. They robustly produce pro-inflammatory cytokines (IL-6, IL-12, IL-1β, and TNF-α) and chemokines (CCL2, CCL5), reflective of their ability to recruit other immune cells to the site of inflammation/tissue damage [24, 34]. Conversely, M2 macrophages are known for their role in tissue repair, phagocytosis of dead cells/cell debris and resolution of inflammation. They specifically express cell surface markers such as CD36, CD206/MRC1, and CD163 [32–35]. M2 macrophages secrete cytokines IL-1RA, IL-10, TGF-β and VEGF [30–32].

In our analysis, ovarian MDMs expressed higher levels of specific marker genes that are unique to M1 phenotypes indicating their involvement in pro-inflammatory response, while TRMs displayed relatively higher levels of CD163, MRC1, CD209, CD200R and IGF1 compared to MDMs, resembling M2-like macrophages previously described in a skeletal single-cell atlas [31]. Previous studies have shown that TRM populations express higher levels of M2 macrophage markers that are mainly involved in tissue repair and resolution of inflammation [32–35]. We also found that NLRP3 inflammasome pathway activity and pyroptosis related genes were significantly enriched in MDM subsets suggesting pyroptotic nature of this specific macrophage subpopulation in the ovary. Interestingly, increased enrichment of pro-inflammatory M1-type macrophages/MDMs showing pyroptotic activity have been noticed in the ovaries of aged women and in PCOS condition [28, 39]. We then compared IHC localization of these two phenotypes (MDMs/TRMs), which showed a marked increase in MDM subpopulation in the ovarian stroma of CG samples compared to controls. CD163+ staining revealed the presence of larger clusters of TRMs in CG when compared with that of control ovaries, which may indicate increased phagocytic activity involved in the removal of dead/damaged cells. A recent study using single-cell atlas of human ovaries revealed the specific role of pyroptotic macrophages in ovarian aging, where the authors indicated that the accumulation of pyroptotic MDMs is responsible for the pro-inflammatory microenvironment, impacting follicle development in the middle-aged group [28]. In a recent review, several studies have indicated that in the early stage of ovarian aging, the M1 type macrophage subset is dominant and plays a pro-inflammatory role contributing to follicular depletion [23].

Next, we explored the specific functional pattern of DEGs in the macrophage cluster between the CG and control groups using IPA enrichment analysis. Functional enrichment analysis revealed that several immunemodulatory and inflammatory response pathways such as IL-1/6/8/12/17 signaling, chemokine signaling, interferon signaling, TNFR1 signaling, antigen presentation pathway, CSF1 signaling in myeloid cells were significantly upregulated in the CG group. AUCell activity scores for the biological processes “regulation of TNF production” and “macrophage activation” was higher in the macrophages of CG group than in the control group. Furthermore, ST data from macrophages showed that NLRP3 inflammasome pathway and IL6-JAK-STAT3 pathway activity scores were higher in CG compared to control samples. The IL-6/JAK/STAT3 pathway plays a crucial role in chemokine-induced inflammatory responses. Chemokines are chemotactic cytokines that regulate immune cell trafficking and function, playing a central role in the immune response by directing the migration and positioning of immune cells including macrophages within tissues [32–34]. Here, we found that several chemokines and some of their receptors such as *CCL2*,* CX3CL1*,* CXCL8/10/12/14*,* CCR2* and *CXCR4* were upregulated in the CG group. Most of these chemokines are known to induce pro-inflammatory responses by recruiting immune cells including macrophages. Moreover, gene expression analysis in the MDM cluster revealed increased expression of pro-inflammatory cytokines and mediators in CG compared with control samples. Notably, key genes associated with the NLRP3 inflammasome and the pyroptotic inflammatory response were upregulated in the MDMs of the CG ovary, suggesting that the macrophage-induced robust inflammatory response may contribute to inflammation and pyroptotic cell-death of follicles, which is further supported by TNF-α and cleaved-CASP-1 expression in the CG group. Indeed, the NLRP3 inflammasome is a multimeric protein complex, that when activated, triggers both inflammation and pyroptotic cell death [40, 41]. Oxidative stress can activate the NLRP3 inflammasome, leading to the release of pro-inflammatory mediators and potentially exacerbating inflammation involving macrophages [41, 42].

Macrophages play a crucial role in oxidative stress-induced inflammation as a source of ROS production [43]. Macrophages have been also known to participate directly in NLRP3 inflammasome-induced pyroptotic cell death thereby releasing pro-inflammatory cytokines (IL-1β and IL-18) that can lead to chronic inflammation and tissue damage [40, 41]. Recent studies have shown that inflammatory aging plays a significant role in the pathogenesis of POI [42, 43]. It has been indicated that oxidative stress and NLRP3 inflammasome interact intricately in the ovarian milieu, significantly affecting oocyte competence and reproductive outcomes [42]. Moreover, it has been reported that the expression of NLRP3 increased in the ovary with age in a mouse model [44]. Increased expression of NLRP3, caspase-1, and IL-1β was also observed in granulosa cells from patients with diminished ovarian reserve exhibiting accelerated ovarian aging [45].

It has been well documented that D-galactose and its metabolites induce increased production of reactive oxygen species in mouse ovaries causing oxidative stress and contributing to apoptosis of granulosa cells [13–15]. The GALT-KO mouse model has often failed to replicate the severe, early-onset POI seen in human classic galactosemia. Although, mouse ovary culture model with D-galactose exposure is useful for studying downstream oxidative stress and to assessing inflammasome pathway activation, however it does not completely mimic the congenital, genetic, and developmental aspects of classic galactosemia caused by GALT deficiency in humans. Therefore, to further corroborate our transcriptomic data, we treated mouse whole ovary cultures with 50 mM D-galactose which is known to induce gonadotoxicity causing POI in animal models. Here we found that D-galactose treatment resulted in oxidative stress in the ovary as evidenced by increased 8-OHdG and 4-HNE expression in the ovarian follicles and stroma. We also found an increased expression of NLRP3, cleaved-CASP1, cleaved GSDMD, cleaved IL1-β and TNF-α in the D-galactose treated ovaries suggesting activation of the inflammasome pathway and pyroptotic cell-death, aggravating the inflammatory microenvironment and likely contributing to follicular atresia.

Additionally, we examined the changes in the transcriptional profiles of ovarian ECs which were characterized into vascular ECs and lymphatic ECs. ECs act as gatekeepers for immune cell trafficking, regulating their extravasation from the bloodstream into the tissue parenchyma for immune homeostasis and during inflammation in several diseases [46–48]. This process involves changes in the expression of several adhesion molecules (VCAM1, ICAM1, selectins, integrins) and cytokines/chemokines (CCL2, CXCL12, CX3CL1, CCL21) by ECs [46–48]. EC activation can be induced by cytokines (IL-6, IL-1β and TNF-α), which then actively recruit immune cells [46–48]. Functional enrichment analysis of DEGs associated with ECs revealed that pathways including cytokine storm signaling, leukocyte extravasation signaling, IL-6, IL-8, and IL-33 signaling were significantly activated in CG. Furthermore, we discovered increased expression of cell-adhesion molecules such as *VCAM1*,* ICAM1*,* PECAM1*,* CLND5*,* SELE*,* SELP* and chemokines (*CCL21*,* CXCL14*,* CX3CL1*,* ACKR1*) in the ECs of CG. These immune modulators play a central role in regulating immune cell trafficking, including diapedesis (trans-endothelial migration) and recruitment of immune cells into the surrounding tissues at sites of inflammation or injury [46–50].

Finally, the cell-cell communication analysis using CellChat tool also unveiled a multitude of signaling pathways associated with immune regulation and inflammatory response being altered in CG versus control. At first, we noted a greater number of cell-cell interactions (ligand-receptor pairs) and increased strength communication among major cell types in the CG group compared with the control group. Next, we examined the relative information flow of signaling pathways among different cell types between the two groups. We identified distinct signaling immune related pathways (GALECTIN, CSF, TAFA, ICAM and IL-16) that were unique to CG groups. Most of these above-mentioned signaling genes regulate macrophage induced inflammatory response. For example, galectin-3 and CSFs play a crucial role in regulating macrophage differentiation, polarization and function by influencing overall pro-inflammatory responses [51–53]. IL-16 promotes the polarization of macrophages towards a pro-inflammatory phenotype and enhances macrophage phagocytic activity [54]. Several signaling pathways such as MIF, IGF1, FN1, CD99, MHC-II, TGF-β, CHEMERIN and VEGF that have been previously reported [55–58] to regulate macrophage-mediated inflammatory immune responses by influencing macrophage activation, migration and recruitment were highly enriched/enhanced in the CGgroup. We also found that PECAM1, ICAM, NCAM, NECTIN and ANNEXIN pathway signals were strengthened in ECs of the CG group. These signals are mainly associated with cell migration and vascular permeability [59], which suggest that endothelial permeability and function have altered in the CG ovary to facilitate immune cell recruitment into the ovarian stroma as evident from the functional enrichment and upstream regulator analyses.

## Conclusions

In summary, we presented a comprehensive investigation of the ovarian immune landscape in prepubertal girls diagnosed with CG using snRNA-seq paired with spatial transcriptomics and identified several cellular and molecular dynamics that may explain the early onset of POI. Our transcriptomic data revealed ovarian heterogeneity, identified four subtypes of macrophages, and highlighted various cytokines/chemokines involved in key immune regulatory pathways associated with proinflammatory immune response. Our IHC data showed increased accumulation of MDM population in the CG ovary that are pro-inflammatory and pyroptotic in nature as evident from specific immune-related gene expression profiles of macrophage subsets. We also elucidated distinct changes in the transcriptomic profile of endothelial cells with respect to cell-adhesion molecules and chemokines/chemokine receptors that may play a crucial role in modulating the immune response in the ovarian stroma. Our ex-vivo experiments on whole mouse ovaries suggest that D-galactose toxicity-induced oxidative stress can activate the NLRP3 inflammasome, initiating a cascade of events involving pyroptosis, and contributing to a pro-inflammatory microenvironment, which adversely impacts the ovarian follicles in the CG condition (Fig. 9 ). Furthermore, the activation of the inflammasome pathway in the CG ovary may trigger a shift in macrophage subsets toward pro-inflammatory MDMs which can lead to a vicious cycle of inflammation. Collectively, our study provides key insights into the transcriptomic profiles of distinct ovarian macrophage subpopulation and the activation of NLRP3 inflammasome pathway involved in the proinflammatory microenvironment which likely contributes to follicular atresia in CG patients, which may lay out future therapeutic interventions to preserve ovarian function. In future studies using an in-vivo mouse model of D-galactose toxicity-induced POI, we would like to address the recruitment of specific macrophages to the site of inflammation and their direct involvement in promoting accelerated follicular atresia in the ovary.

### Limitations of the study

Ovarian tissue cryopreservation provides an opportunity to obtain tissue biopsies in small quantities for evaluation which would normally not be ethical to collect in young individuals (prepubertal girls). As CG is a rare metabolic disorder affecting young children, we are limited not only by the number of patients [age group 5–7 years] who chose this experimental option (both those with classic galactosemia and those with solid tumors as controls) but also by the quantity of tissue allocated for research. Our ex-vivo mouse whole ovary model shed light into the D-galactose toxicity-induced oxidative stress, which promotes activation of the NLRP3 inflammasome, initiating a cascade of events involving pyroptotic cell death of ovarian follicles, which is likely contributing to accelerated follicular atresia, and increase in the pro-inflammatory macrophage population in the ovary. However, further investigation is needed to fully establish the direct role of pyroptotic macrophages [MDMs] in triggering POI condition in CG. Future studies using a galactose-induced POI mouse model may provide further details on whether the pro-inflammatory transition of macrophages in the ovary contributes to follicle loss and POI progression.

## Supplementary Information

Supplementary Material 1.

Supplementary Material 2.

## Data Availability

The snRNA-seq and spatial transcriptomics data that support the findings of this study were deposited in the Gene Expression Omnibus (GEO) repository with accession number GSE267932 and GSE308275.
