# Supplementary material for: Transcriptomic profiling of the ovarian immune landscape reveals distinct macrophage subsets and activation of the NLRP3 inflammasome likely contributing to accelerated follicular atresia in classic galactosemia
Source: Cell Commun Signal. 2026 May 30;24:428. doi: 10.1186/s12964-026-02950-9 (PMC13439996; doi:10.1186/s12964-026-02950-9)
Supplement: Supplementary file 1 [file 12964_2026_2950_MOESM1_ESM.pdf]

Supplementary Table S1

Demographics of 6 patients used in this study for snRNA-seq and ST analysis

Patient #	Diagnosis	Age (year)	Genotype	Primordial follicle count	Mean Follicle density (follicles/mm^2)
CG3	Classic Galactosemia	5	Q188R/Q188R	20	7.9
CG4	Classic Galactosemia	7	R148W/R231C	40	6.0
CG5	Classic Galactosemia	7	Q188R/Q188R	98	10.4
	Controls				
C4	Rhabdomyosarcoma	7	N/A	191	14.4
C5	Medulloblastoma	6	N/A	131	12.4
C6	Medulloblastoma	6	N/A	223	24.7
