# Supplementary material for: Transcriptomic profiling of the ovarian immune landscape reveals distinct macrophage subsets and activation of the NLRP3 inflammasome likely contributing to accelerated follicular atresia in classic galactosemia
Source: Cell Commun Signal. 2026 May 30;24:428. doi: 10.1186/s12964-026-02950-9 (PMC13439996; doi:10.1186/s12964-026-02950-9)
Supplement: Supplementary file 2 [file 12964_2026_2950_MOESM2_ESM.pdf]

Supplementary Figures

Figure S1

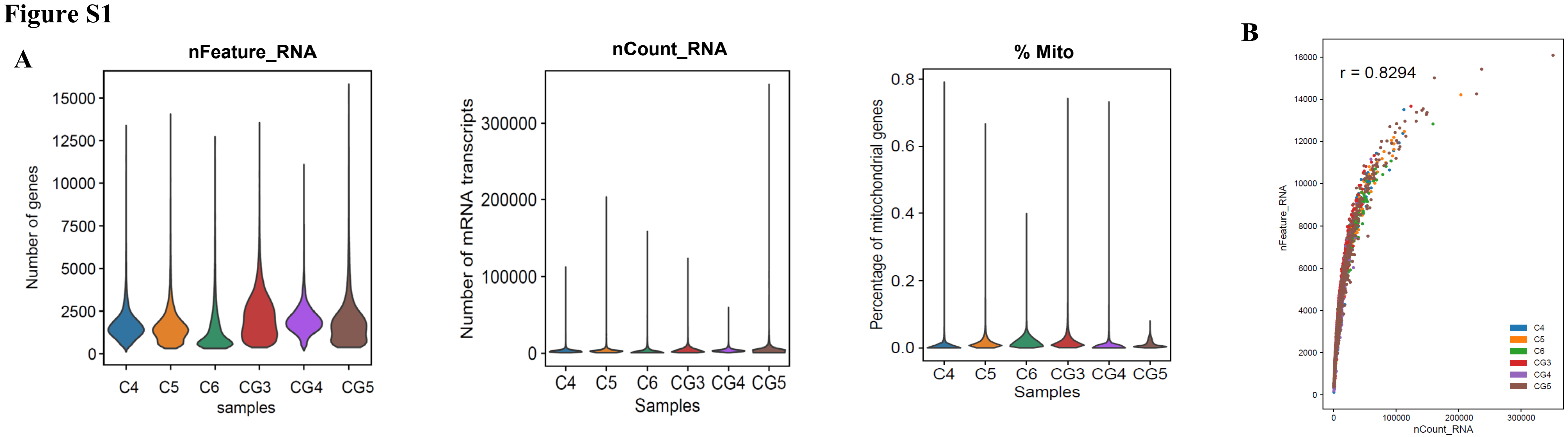

Figure S1: Quality control and integration of snRNAseq data. A) Violin plots showing the number of genes, RNA counts, and percentage of mitochondrial genes of each single-cell transcriptome sequencing sample. (B) Pair-wised relationship between RNA transcripts, and number of genes per sample.

Figure S2

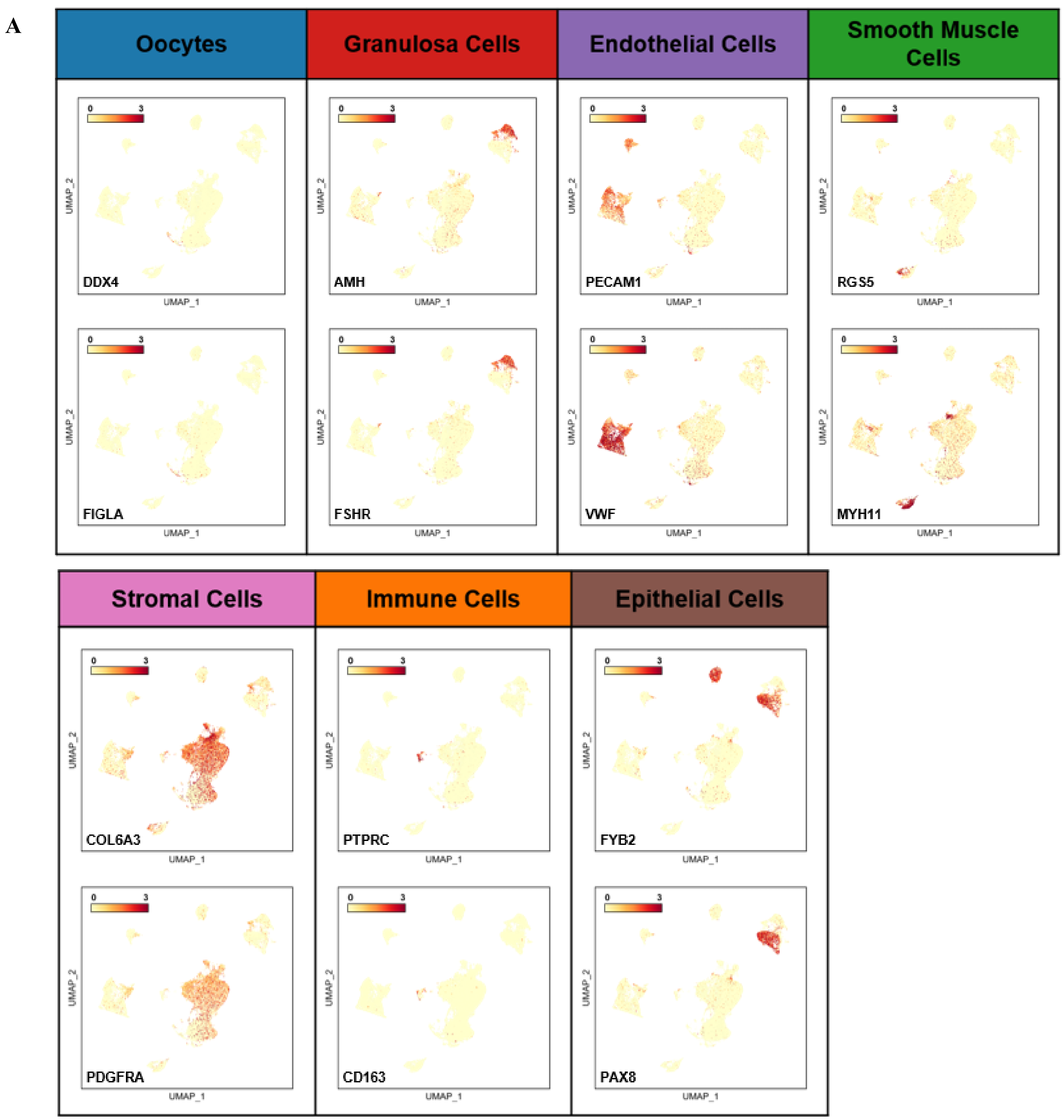

Figure S2: A) UMAP plots showing expression of canonical marker genes used to identify seven major ovarian cell types.

Figure S2

B

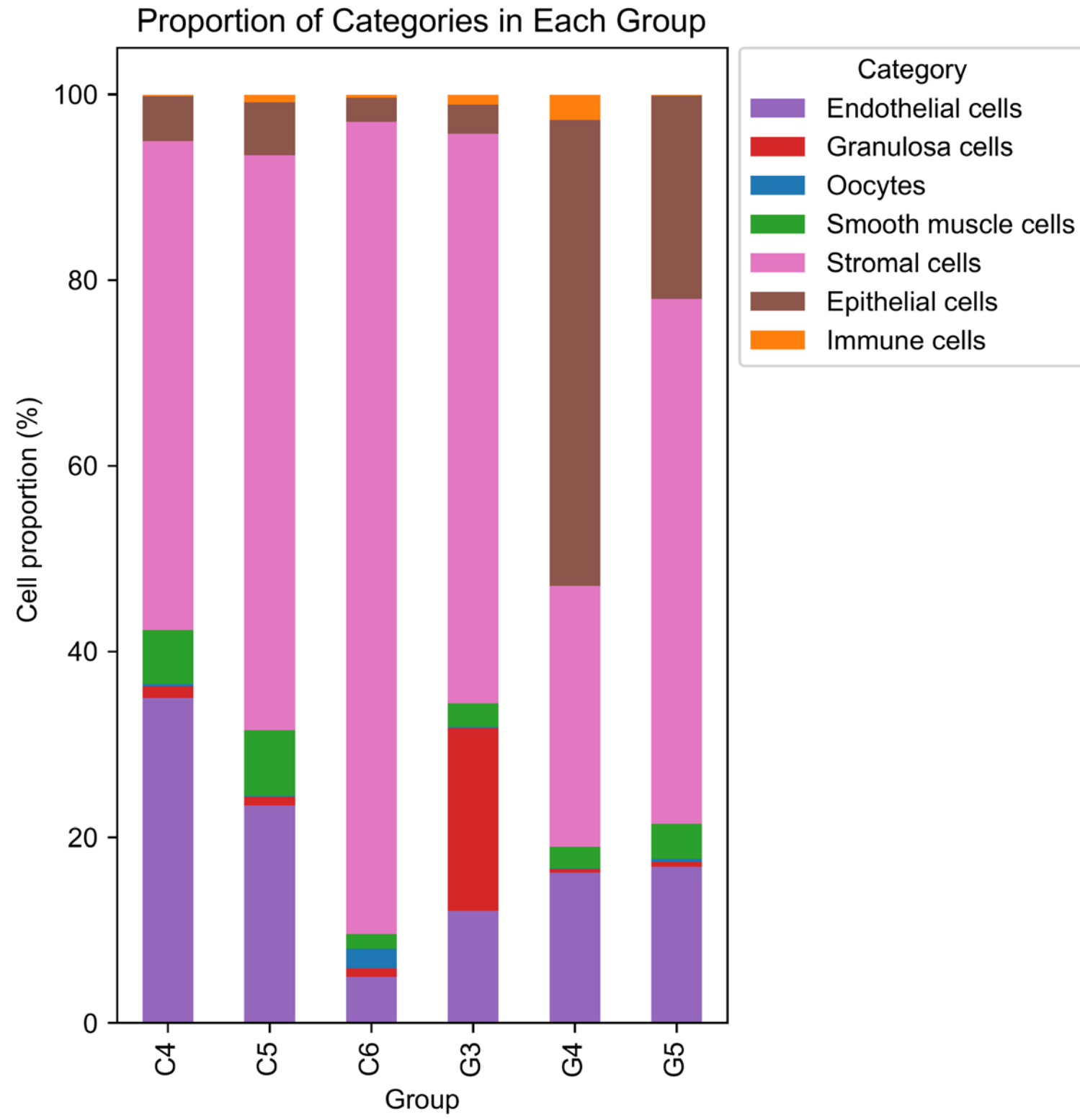

C

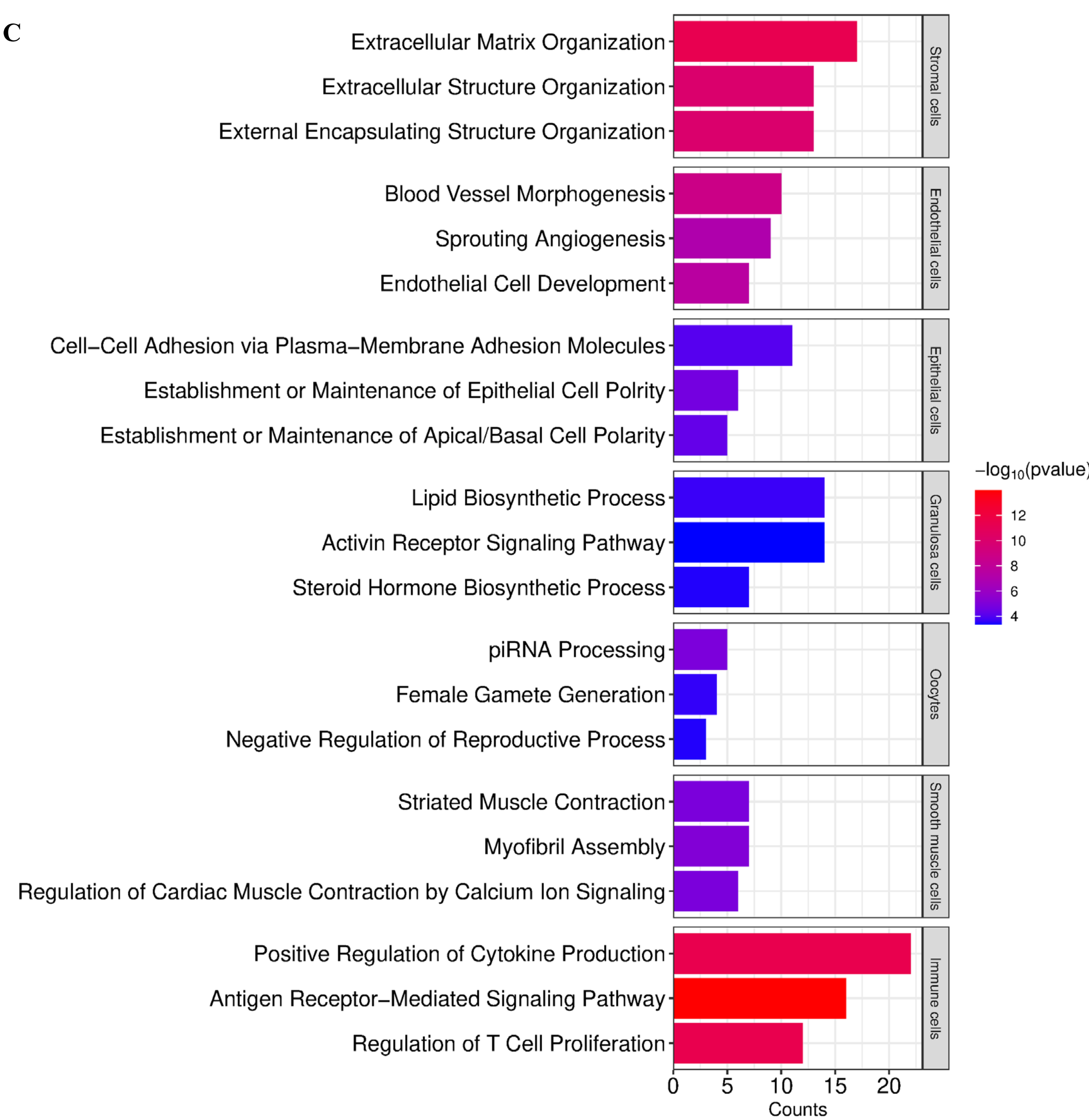

B) Gene ontology analysis showing biological processes associated with highly expressed markers found within each cell type cluster. C) Bar graph showing percentage composition of 7 major cell types in all the six patient's ovary samples.

Figure S3

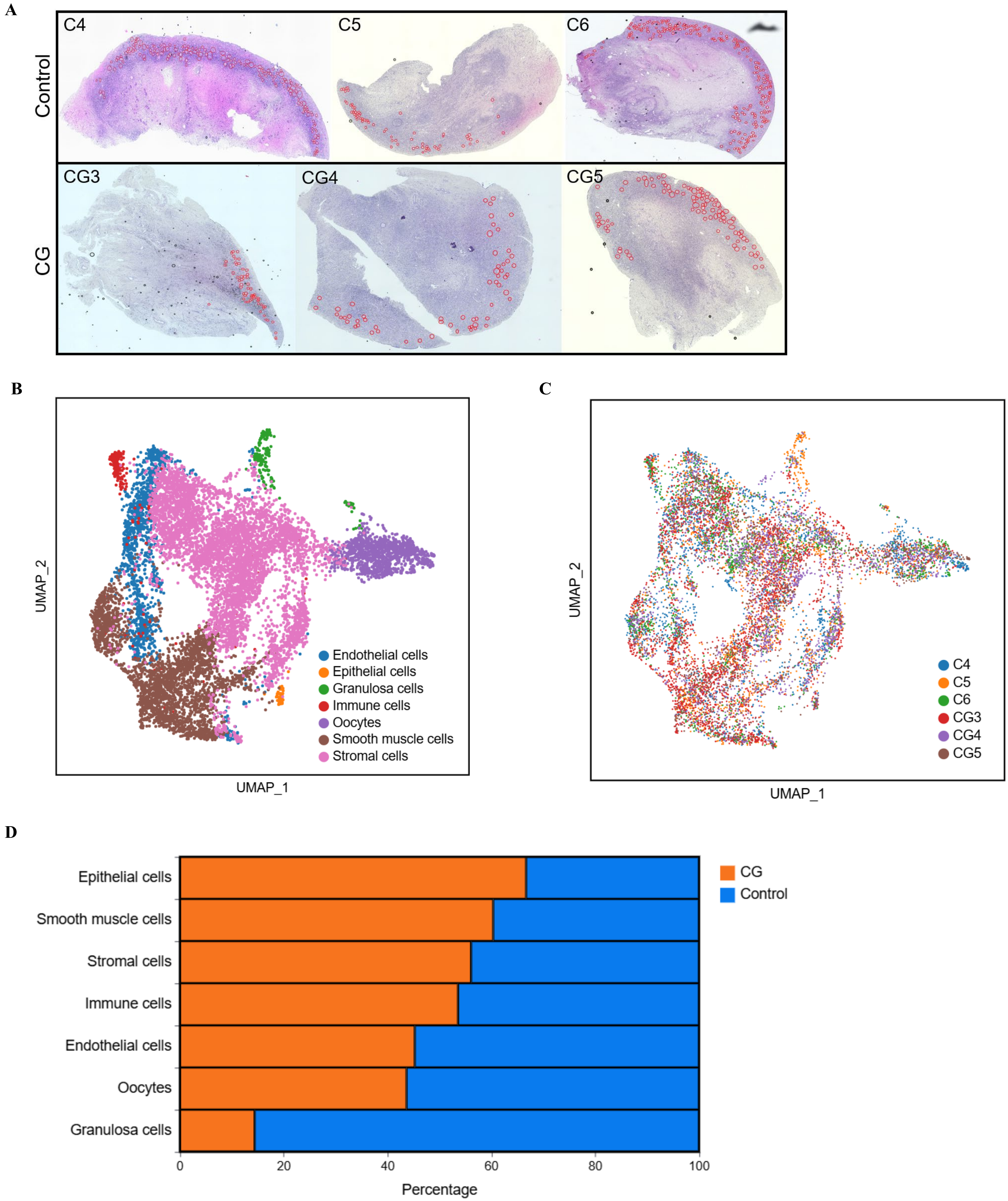

Figure S3: A) Histology of H&E-stained ovary sections from control and CG patients used for Visium ST experiment. Red circles indicate ovarian follicles. B) UMAP plot showing distribution of 7 major cell types as ST spots. C) UMAP plot showing distribution of ST spots for all the 6 patient ovary samples. D) Violin plot showing some of the representative canonical marker genes for annotating major ovairan cell types in ST data.

Figure S4

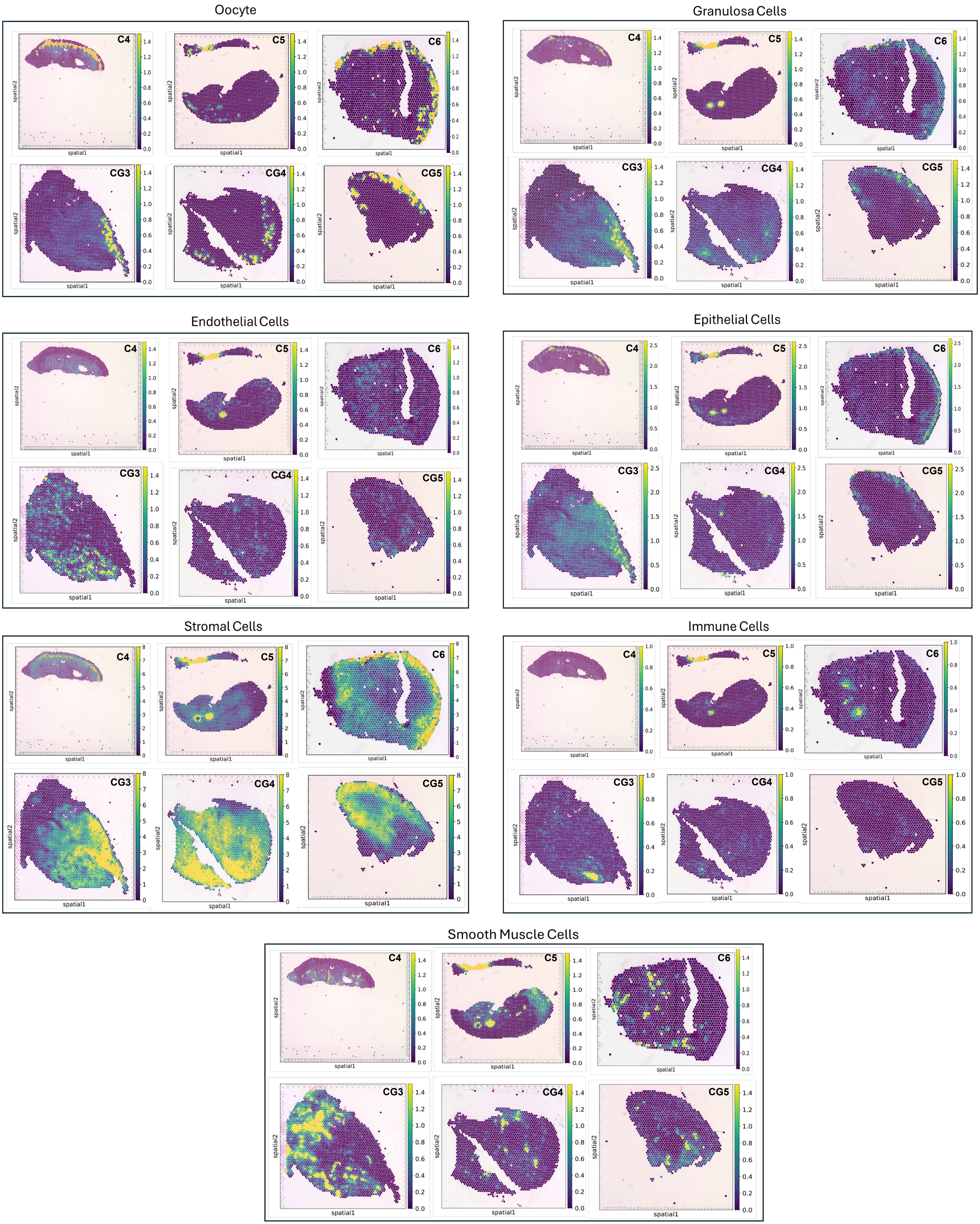

Figure S4: Spatial feature plots depicting the gene expression map of seven major ovarian cell types displaying spot-based enrichment of reference cell types from snRNAseq data.

Figure S5

A

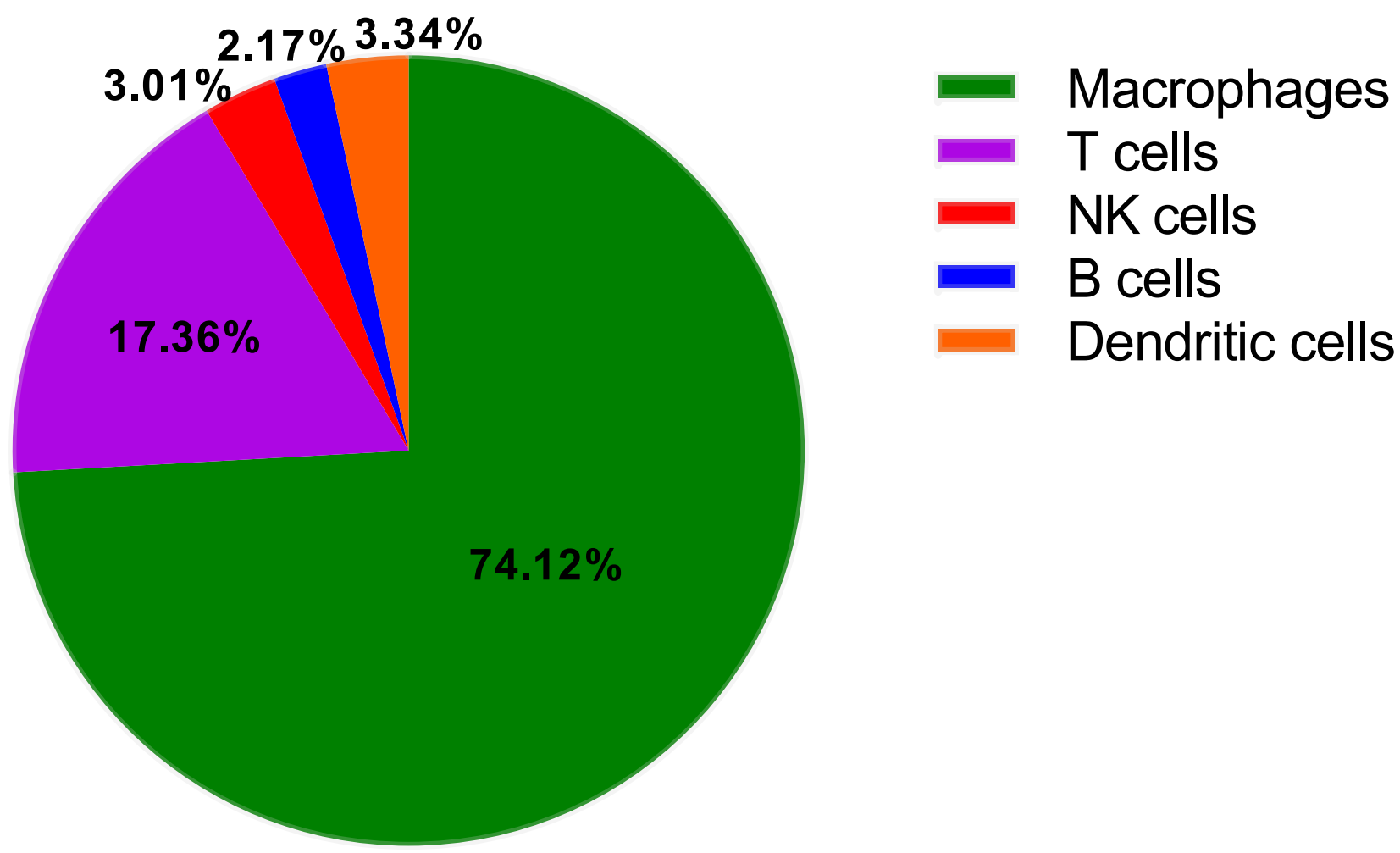

B

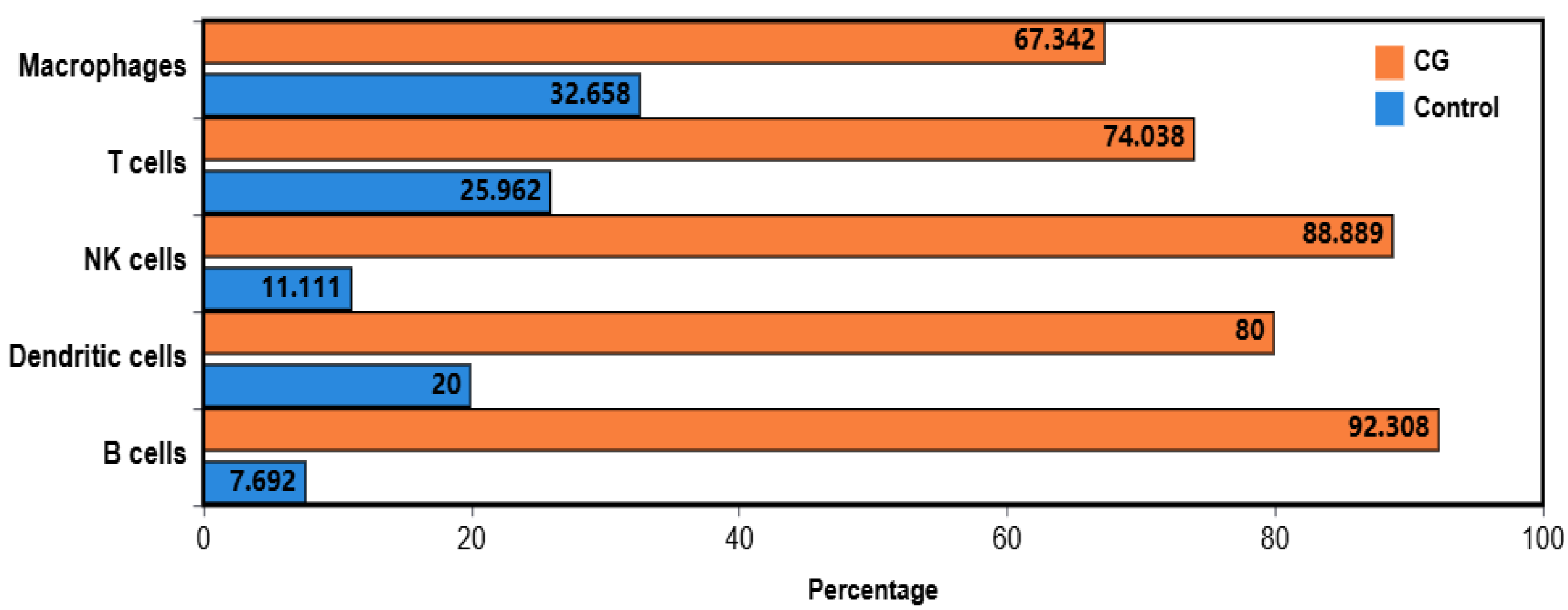

Figure S5: A) Pie chart depicting the overall percentage composition of different immune cells from all the ovary samples taken together indicating macrophages are the major immune cells in the human ovary atlas. B) Bar plot showing differences in cell proportion of five different immune cell types between CG and control samples.

Figure S6

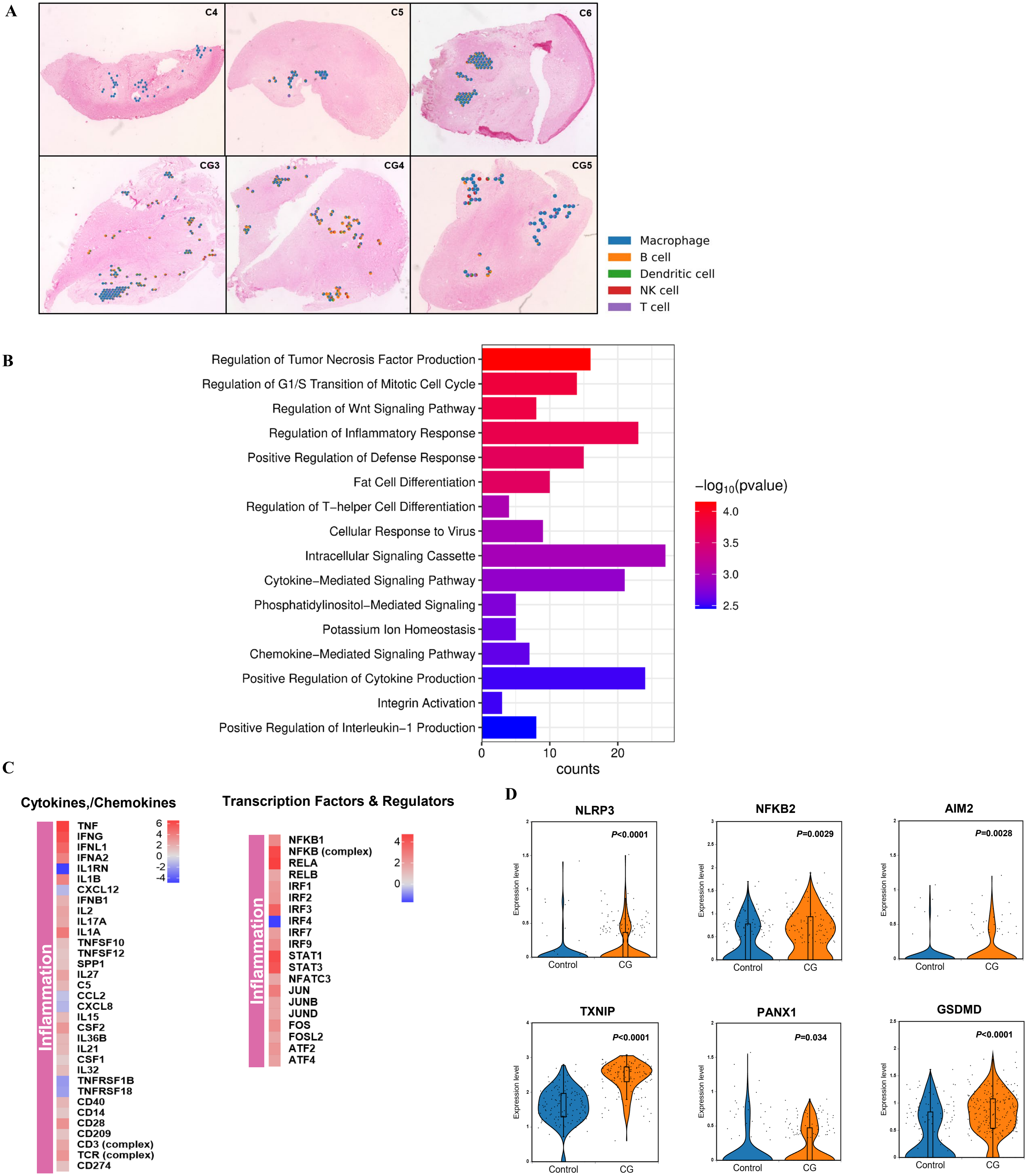

Figure S6: A) Spatial feature plots depicting the distribution and proportion of five different immune cells in the CG and control ovary sections based on the reference marker genes from snRNAseq data. B) Gene ontology analysis on DEGs showing biological processes that are significantly enriched in macrophages of CG group. C) IPA upstream regulator analysis on DEGs showing upstream genes that are activated/inhibited (CG vs control, z-score) in macrophages.) Expression levels of *NLRP3, NFKBI1, AIM2, TXNIP, PANX1* and *GSDMD* genes associated with inflammasome pathway. Unpaired *t*-test was used to compare the gene expression levels between CG and control groups showing statistically significant *P* values.

Figure S7

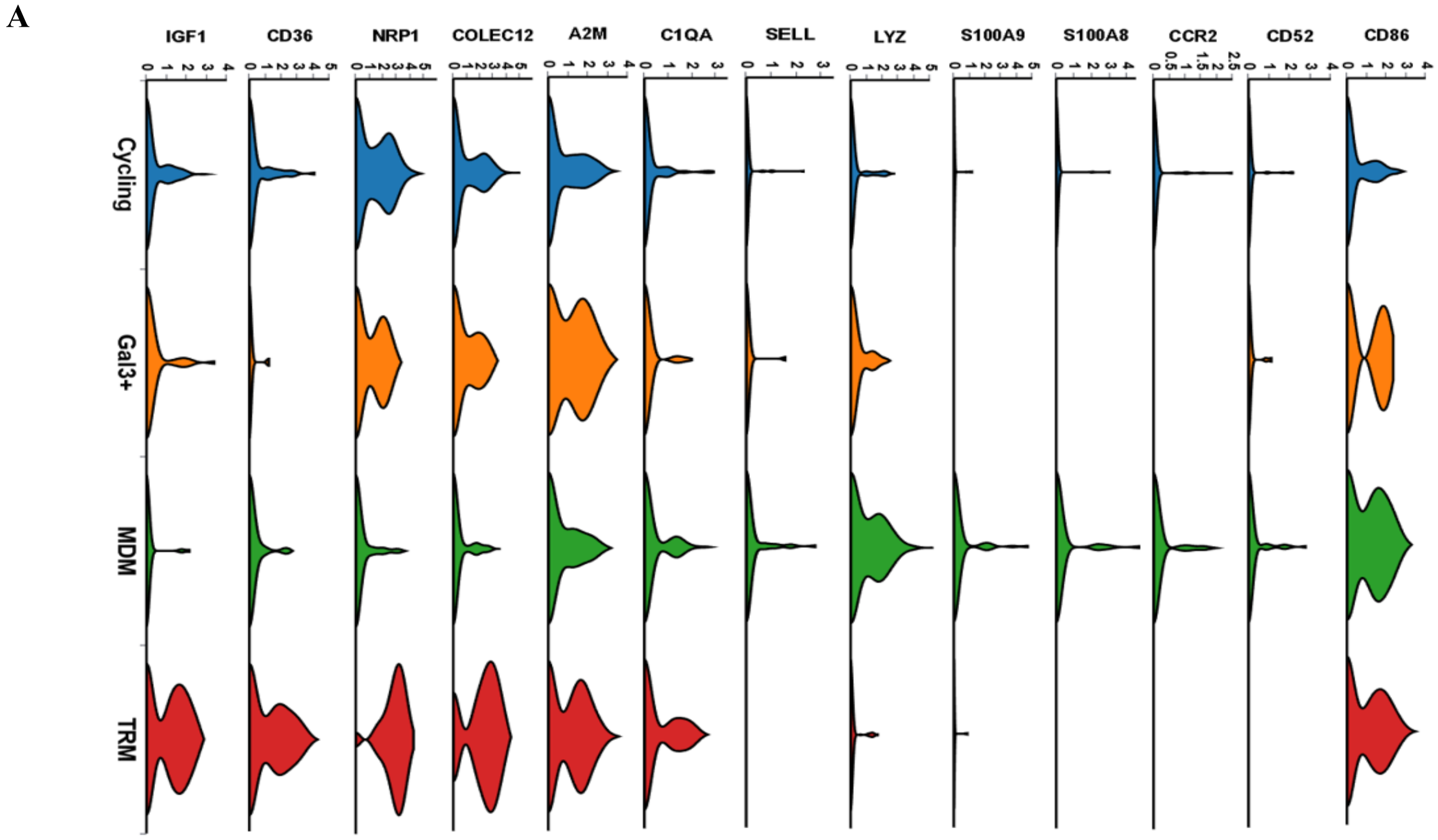

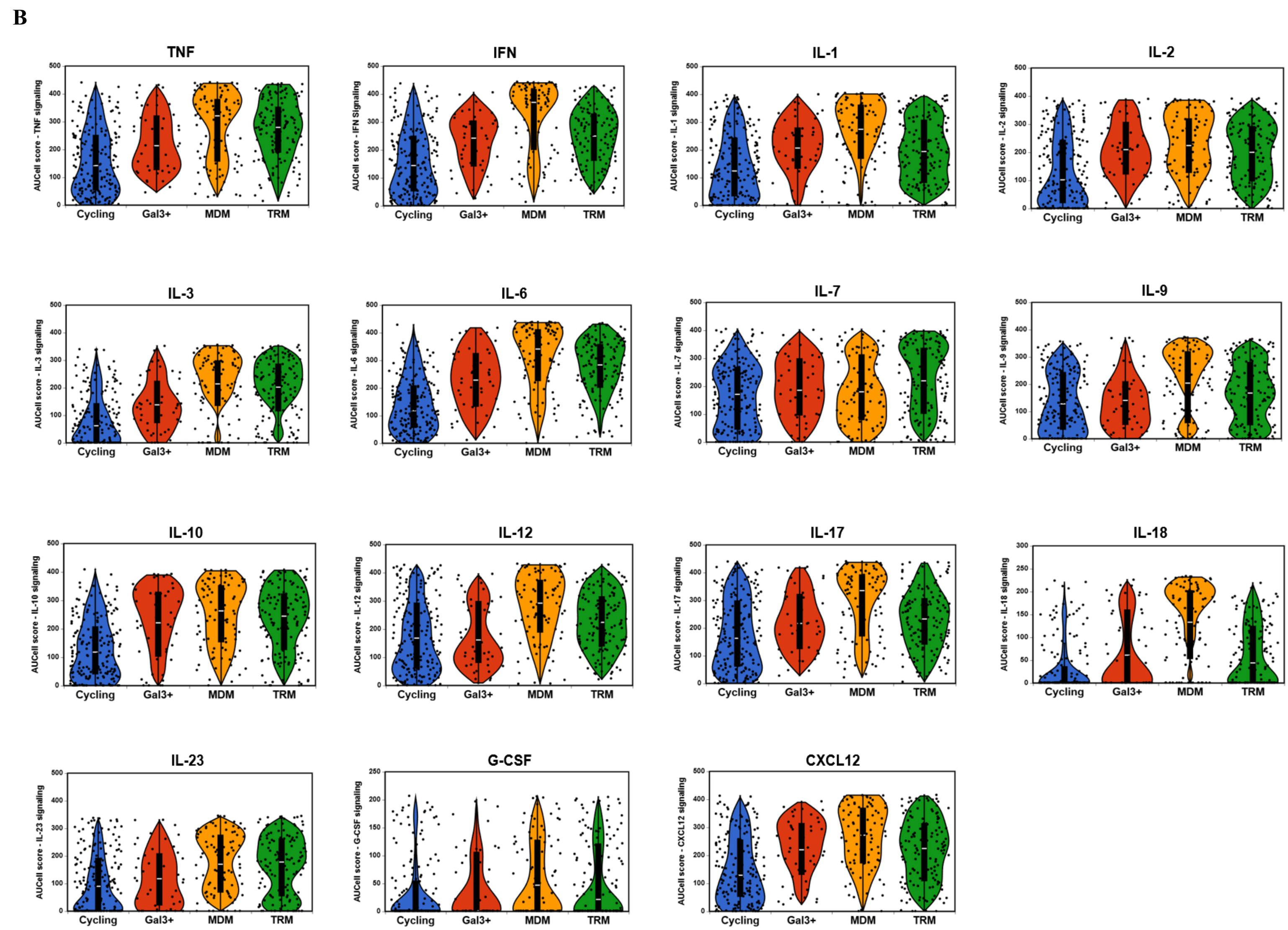

Figure S7: A) Transcriptomic signature genes unique to macrophages identified in 4 different subpopulations of macrophages. B) AUCell activity score of different cytokines in four distinct macrophage subsets.

Figure S7

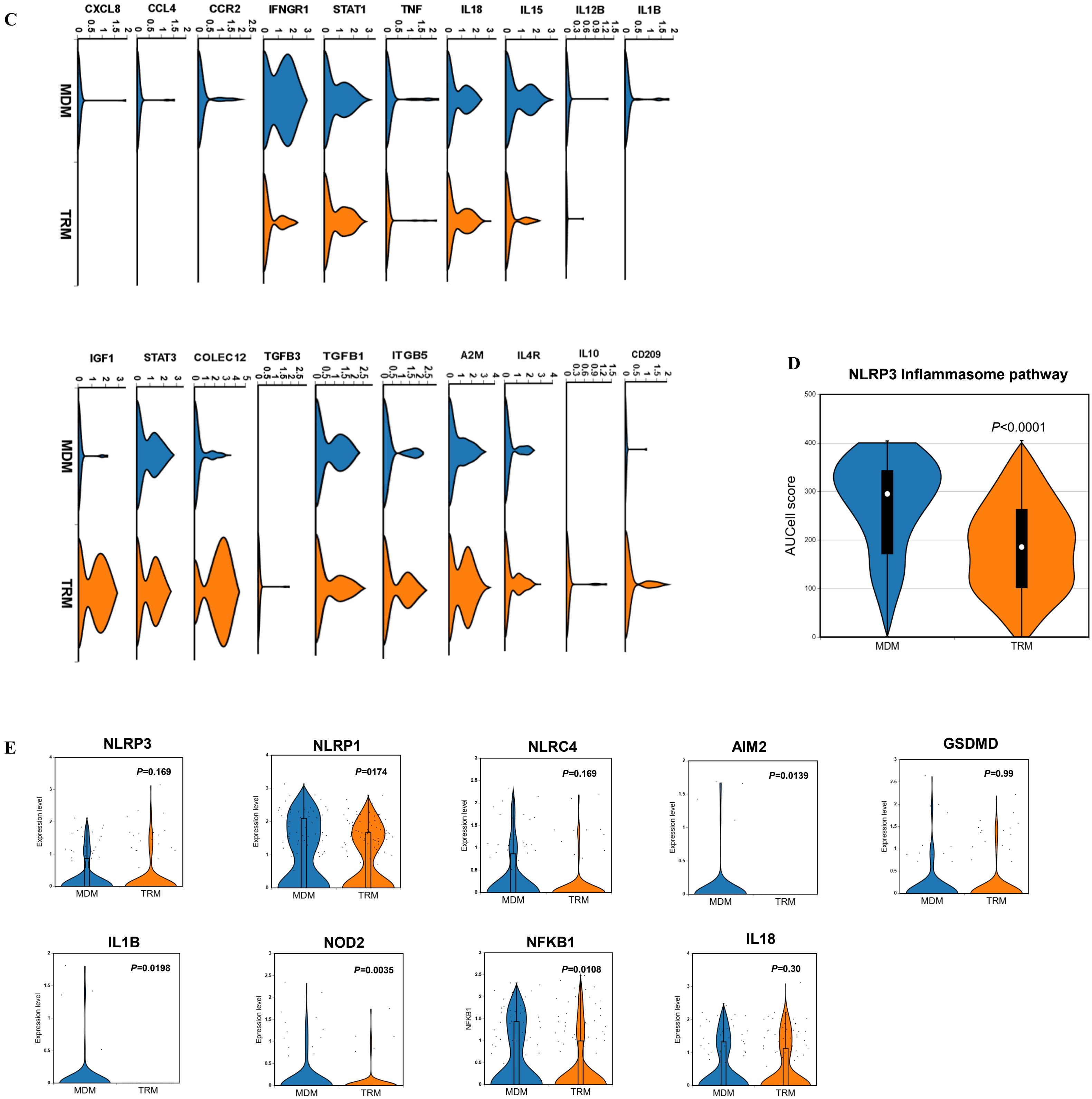

C) Pro-inflammatory (M1-like macrophage) and anti-inflammatory (M2-like macrophage) canonical marker genes identified in MDM and TRM subtypes D) AUCell activity score of NLRP3 inflammasome pathway between MDMs and TRMs E) Expression of key inflammasome pathway genes between MDM and TRM subsets. Unpaired *t*-test was used to compare the gene expression levels between MDMs and TRMs with statistically significant *P* values.

Figure S7

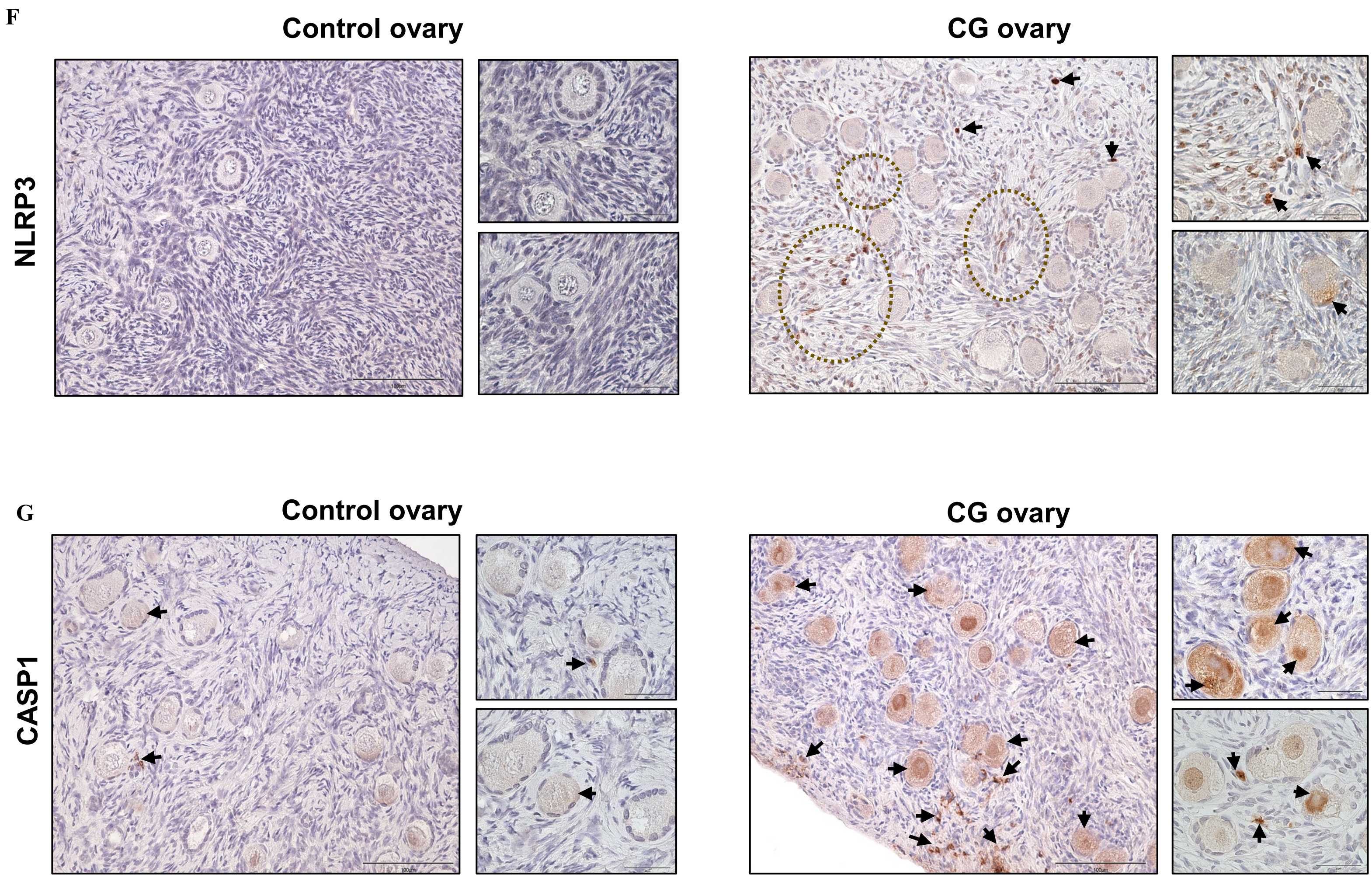

F) and G) Representative IHC images of ovaries showing expression of NLRP3 and CASP1 in CG and control patient group. Higher magnification (right panels) taken at different regions of the ovary section using a 60x oil immersion lens. Arrows indicate IHC DAB signal in the follicles and stromal cells

Figure S8

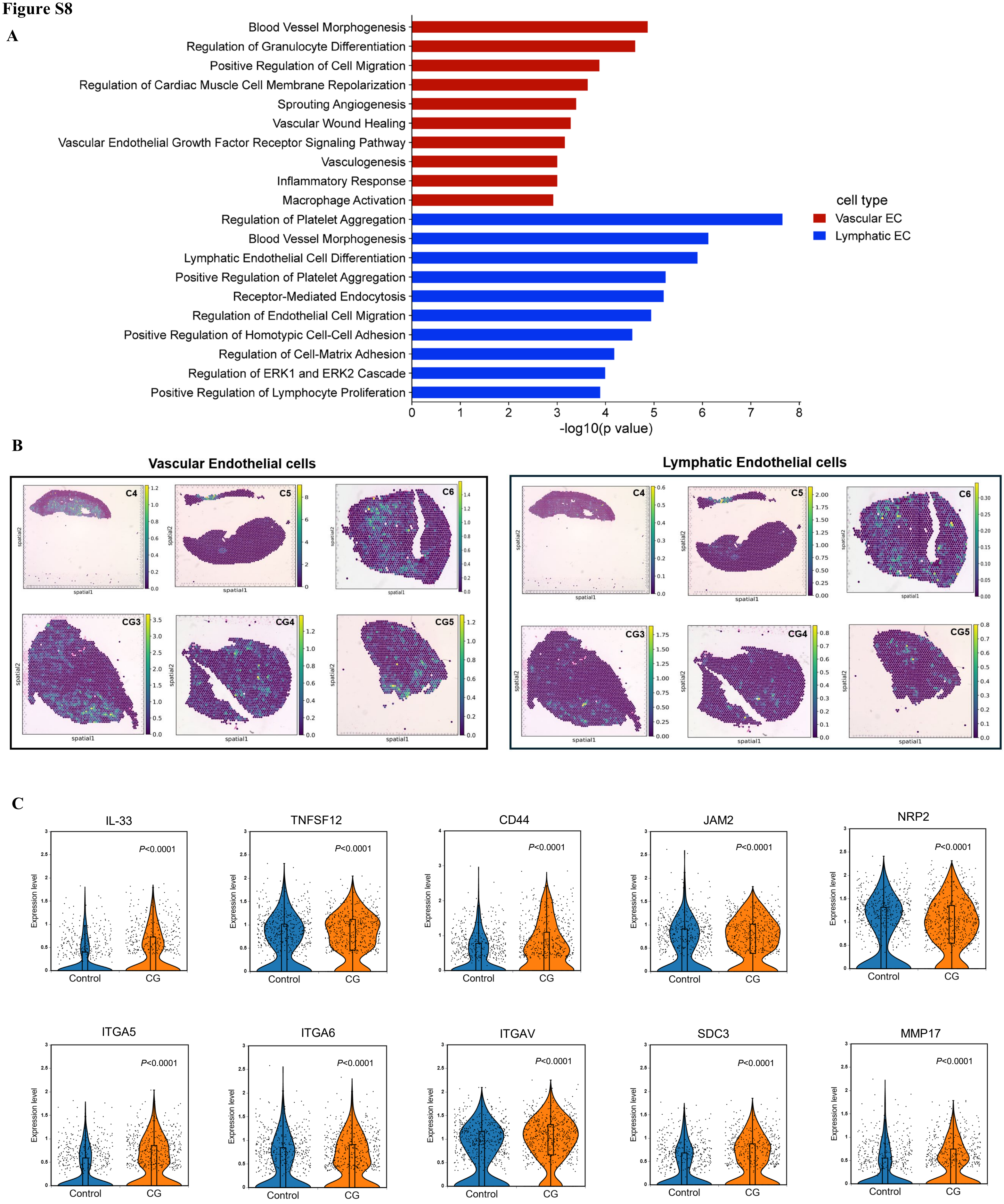

Figure S8: A) GO enrichment plot showing various biological processes associated with the DEGs from ovarian vascular and lymphatic EC subpopulations. B) Spatial feature plots of all the six ovary samples showing mapping of vascular and lymphatic ECs as enriched ST spots based on marker genes. C) Gene expression levels of Immunomodulatory signature genes that influence endothelial cell behavior, vascular permeability and transmigration of immune cells. Unpaired t-test was used to compare the gene expression levels between CG and control groups showing statistically significant P values.

Figure S9

A

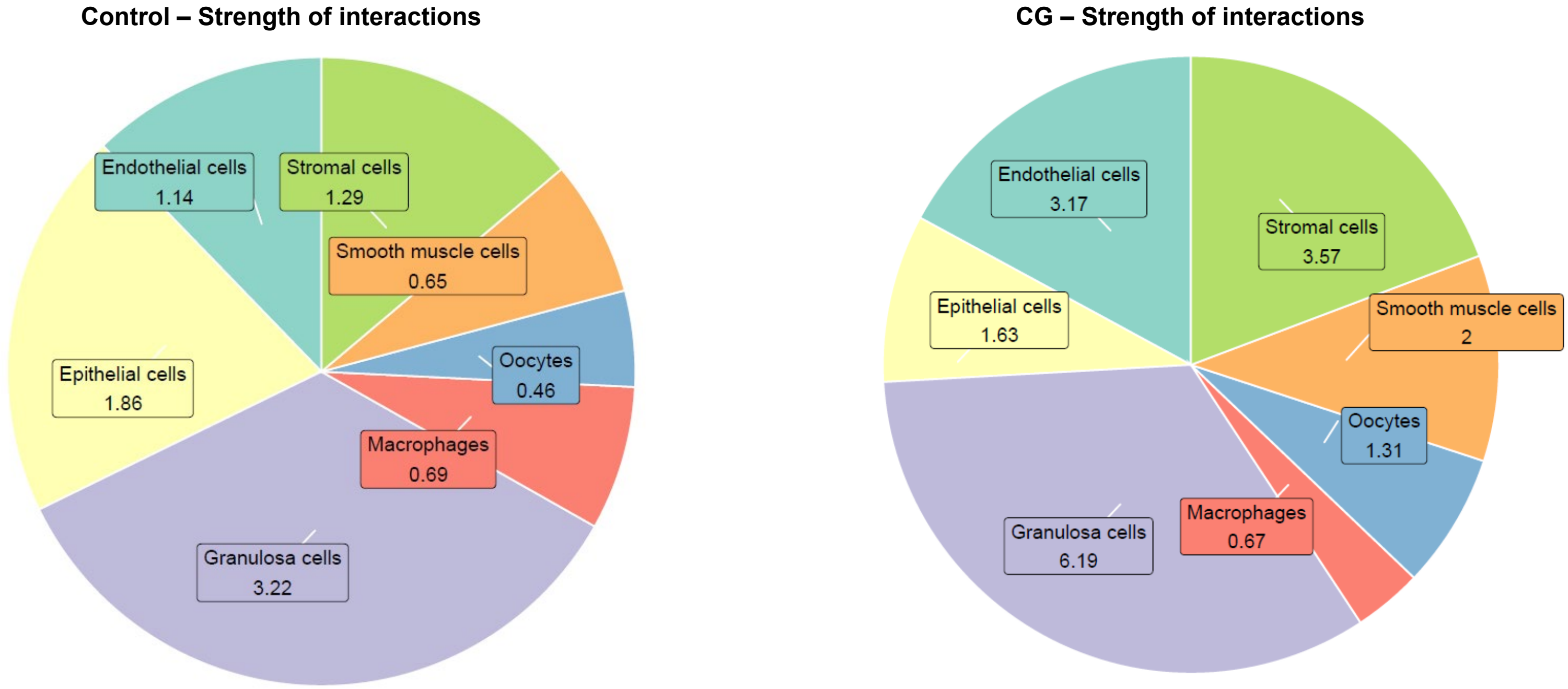

B

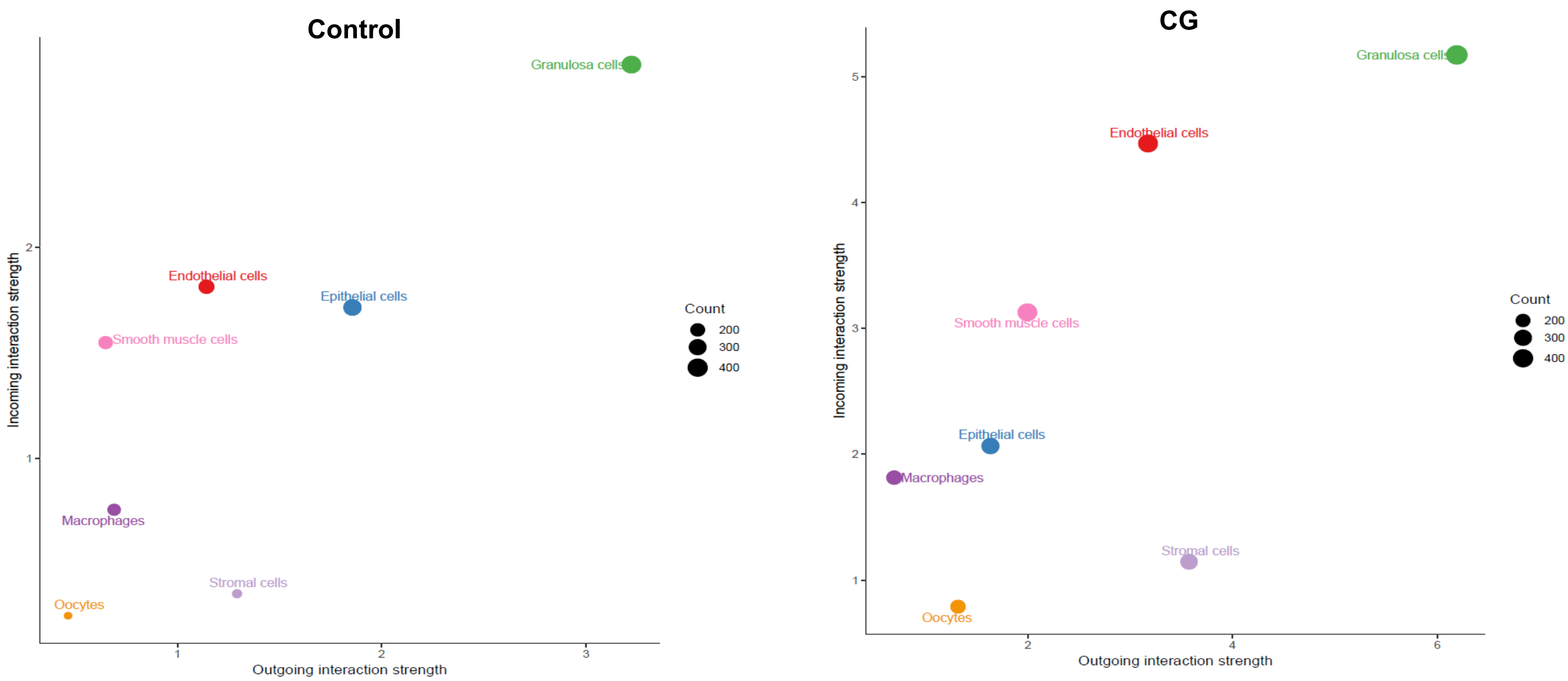

Figure S9: CellChat analysis illustrating cell-cell communication network among different ovarian cells. A) Pie chart depicting the strength of ligand-receptor interactions in 7 major ovarian cell types between CG and control group. B) Scatter plot of incoming and outgoing interaction strength of each cell population in CG and control group.

Figure S10

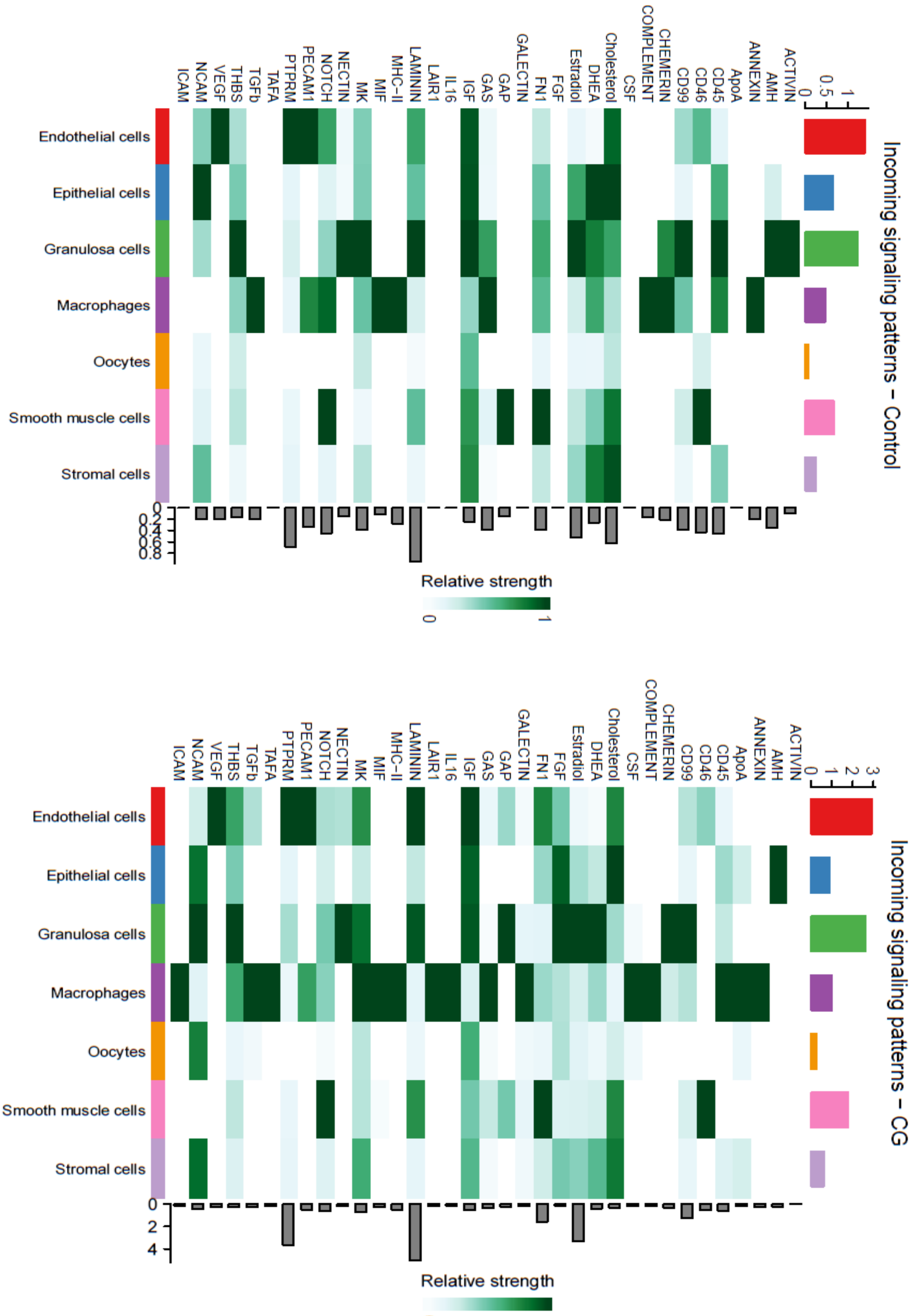

Heatmap showing changes in incoming signaling patterns for each signaling pathway in each cell population between CG and control groups.

Figure S11

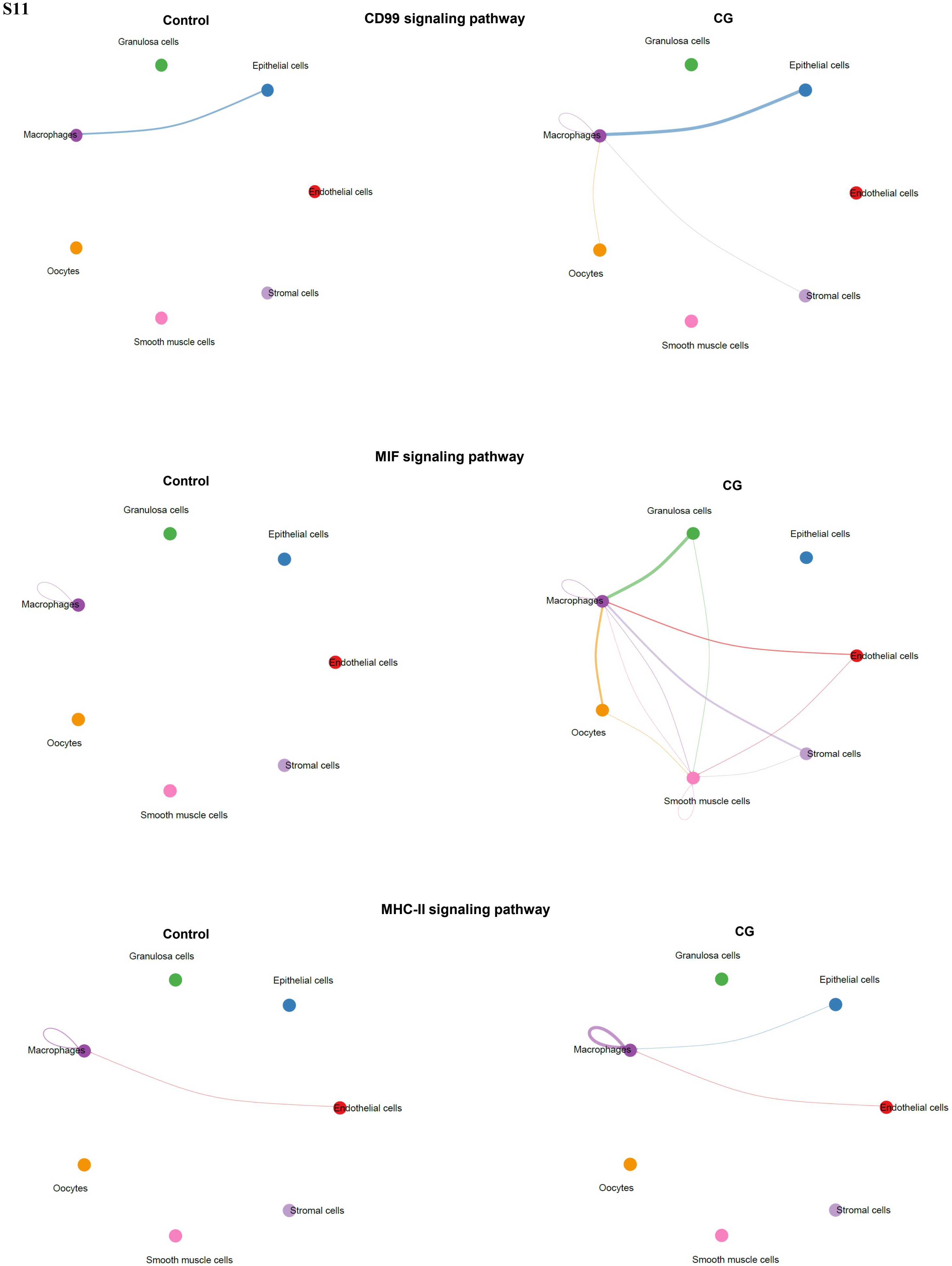

Figure S11: Circle plot showing CD99, MIF and MHC-II signaling pathway networks in CG and control group

Figure S12

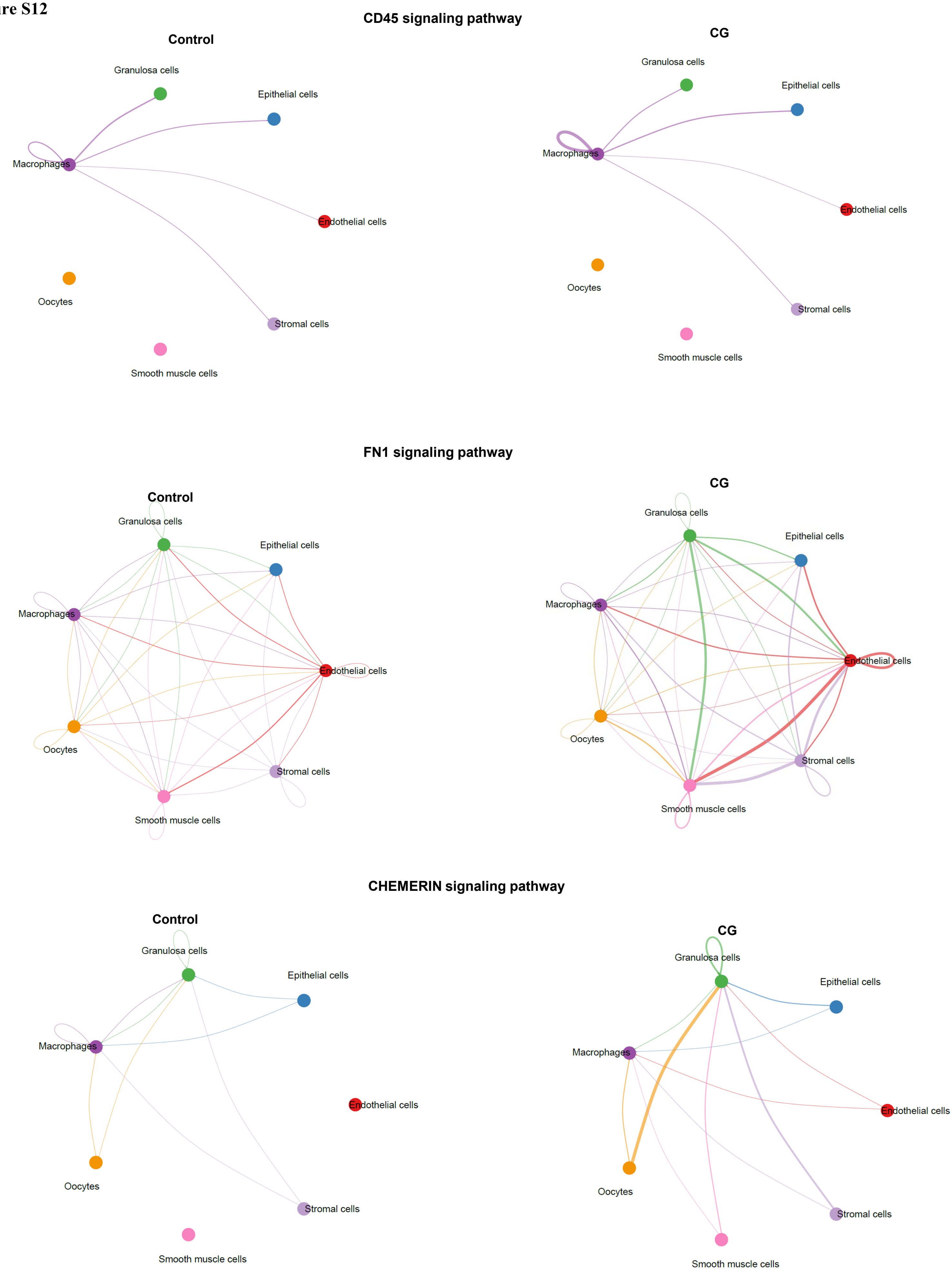

Figure S11: Circle plot showing CD45, FN1 and CHEMERIN signaling pathway networks in CG and control group

Figure S13

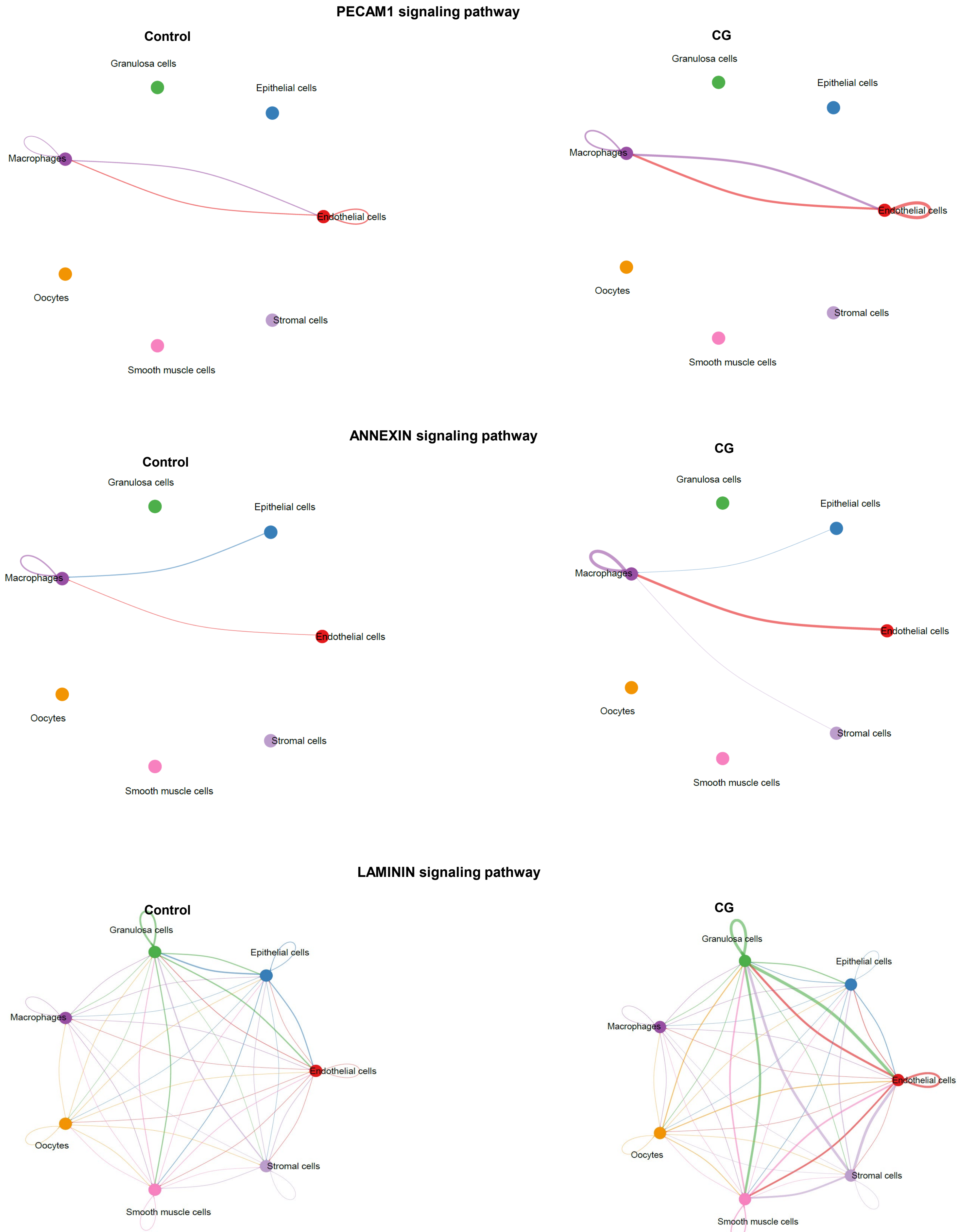

Figure S11: Circle plot showing PECAM1, ANNEXIN and LAMININ signaling pathway networks in CG and control group
